# Sand cat swarm optimization algorithm and its application integrating elite decentralization and crossbar strategy

**DOI:** 10.1038/s41598-024-59597-0

**Published:** 2024-04-18

**Authors:** Yancang Li, Qian Yu, Zunfeng Du

**Affiliations:** 1 School of Civil Engineering, Hebei University of Engineering, Handan, 056038 Hebei China; 2 School of Civil Engineering, Tianjin University, Tianjin, 300354 China

**Keywords:** Sand cat swarm optimization algorithm, Dynamic exponential factor, Elite decentralization strategy, Crossbar strategy, Engineering application, Electrical and electronic engineering, Mechanical engineering

## Abstract

Sand cat swarm optimization algorithm is a meta-heuristic algorithm created to replicate the hunting behavior observed by sand cats. The presented sand cat swarm optimization method (CWXSCSO) addresses the issues of low convergence precision and local optimality in the standard sand cat swarm optimization algorithm. It accomplished this through the utilization of elite decentralization and a crossbar approach. To begin with, a novel dynamic exponential factor is introduced. Furthermore, throughout the developmental phase, the approach of elite decentralization is incorporated to augment the capacity to transcend the confines of the local optimal. Ultimately, the crossover technique is employed to produce novel solutions and augment the algorithm's capacity to emerge from local space. The techniques were evaluated by performing a comparison with 15 benchmark functions. The CWXSCSO algorithm was compared with six advanced upgraded algorithms using CEC2019 and CEC2021. Statistical analysis, convergence analysis, and complexity analysis use statistics for assessing it. The CWXSCSO is employed to verify its efficacy in solving engineering difficulties by handling six traditional engineering optimization problems. The results demonstrate that the upgraded sand cat swarm optimization algorithm exhibits higher global optimization capability and demonstrates proficiency in dealing with real-world optimization applications.

## Introduction

The rapid development of industry and the evolving landscape have given rise to an assortment of engineering applications. Our objective is to enhance the efficiency of these technical challenges within a designated period. Each engineering application presents unique solutions, and it is evident that no singular optimization technique possesses the capability to effectively tackle all optimization challenges effectively. Hence, this proliferation of applications has introduced additional challenges to the field of optimization. Historically, optimization methods have been widely employed across a diverse range of applications, such as path planning^1,2^, location problem^3^, production shop scheduling^4,5^, power generation prediction^6^ and a multitude of additional issues. In the present era, as engineering issues become more intricate and challenging to simulate, it is crucial to prioritize accelerating the creation of superior optimization algorithms. Hence, the process of optimizing algorithms is still far.

Scholars have developed a range of optimization algorithms, drawing inspiration from biology, nature, and society. These optimization techniques have been refined and extensively employed to address diverse intricate engineering challenges. Optimization algorithms can be divided: swarm, evolutionary, physical, and human. Optimization algorithms for populations aim to replicate the social behavior observed in populations. Examples of optimization algorithms that replicate the predatory behavior of creatures include the whale optimization algorithm (WOA)^7^, the Harris Eagle algorithm (HHO)^8^, and the chimpanzee optimization algorithm (COA)^9^. Additionally, the Black tern algorithm (STOA)^10^ emulates the migratory and aggressive life patterns observed in black tern groups. Darwinian evolution inspires for evolutionary optimization techniques. One of the algorithms that falls within this category is the backtracking search optimization algorithm (BSA)^11^ and the differential evolution algorithm (DE)^12^. Physical optimization techniques are derived based on fundamental principles of physics. The principle of simulated annealing is based on the simulated annealing algorithm (SA)^13^. The proposed technique, known as the gravitational search algorithm (GSA)^14^, draws inspiration from the law of gravitation. The method known as the black hole algorithm (BHBO)^15^ is derived from the inherent characteristics of black holes. The algorithms known as human optimization algorithms are derived from the study of human behavior. One instance of an optimization algorithm is the brainstorming optimization method (MSA)^16^, which utilizes of human behavior to address optimization problems. Another algorithm, Human Learning Optimization (HLO)^17^, originated from a simplified model of human learning. Optimization challenges possess a notable capacity to effectively address engineering problems.

With a growing variety of proposed optimization methods, numerous researchers have enhanced these algorithms. An enhanced self-adaptive beneficial factor-based SOS (SaISOS) with adaptive beneficial factors was proposed by Nama et al.^18^. The researcher incorporated a three-way mutualism phase into the model, along with the introduction of a random-weighted reflection coefficient and a novel control operator. In a sequence of tests, the enhanced algorithm demonstrates a significant superiority over its competitors. Nama et al.^19^ introduced a refined backtracking search technique known as GQR-BSA. The client updates the coordinate structure of BSA by implementing quasi-reflection, quantum Gaussian mutation, adaptive parameter execution, and leaping based on quasi-reflection. This permits the system to transition from the local optimal to the global optimal. In their study, Nama et al.^20^ presented an enhanced symbiosis algorithm known as I-SOS. The setting up of a balance between the core of exploration and activity is achieved by employing adjusted return factors, modified parasite stages, and search strategies that rely on random weights. The results of the benchmark function test demonstrate that the implementation of I-SOS translates to a boost in search performance. Nama et al.^21^ blended the SMA algorithm with the quasi-reflectomy-based learning mechanism (QRBL), resulting in the facilitation of population diversity early development, improved convergence, and elimination of local optimizations. Luo et al.^22^ proposed a multi-objective balance optimizer slime mold algorithm (MOEOSMA), which uses dynamic coefficients, an elite filing mechanism, a crowding distance method and an equalization pool strategy to enhance the algorithm's capability. The test findings illustrate MOEOSMA's intense competition. Yin et al.^23^ introduced a multi-objective EOSMA (MOEOSMA). The equilibrium optimizer's concentration update operator is applied. After optimizing the value using the greedy technique, the random difference mutation operator is added. MOEOSMA has a lower solution time and an improved convergence accuracy, according to the equivalent results.

Scholarly improvements to algorithms render them more suitable for complex engineering optimization issues, other than to be applicable to simple examples like trusses. Zhang et al.^24^ put forward a search algorithm for bald eagles based on bionic polar coordinates (PBES). To improve the algorithm, the initialization is modified, and parameters and polar coordinates are introduced. Upon conducting tests, it has been determined that the enhanced algorithm exhibits a favorable impact on the approximation of curves. With the goal to ascertain the active earth pressure of retaining walls supporting C-backfill, for instance, Nama et al.^25^ developed a novel improved backtracking search optimization algorithm (IBSA) based on adaptive control parameters. The findings demonstrated that it had a positive impact. Chakraborty et al.^26^ introduced an enhanced symbiotic search method called NMSOS. It proved that the algorithm improved on all outcomes and could use a multistage threshold method with varying thresholds to segment COVID-19 chest X-ray pictures. The original algorithm's search capability is altered by integrating the development potential of SOS with the searching potential of SQI. This method establishes the shallow strip foundation's seismic bearing capacity under pseudodynamic conditions and prepares it for numerical analysis.

In the current study, a novel swarm intelligence optimization algorithm is chosen. In 2022, Seyyedabbasi et al.^27^ presented the Sand Cat swarm optimization technique. The primary function of this program is to imitate the sand cat's hunting habits. Because it's hard to get food in the desert, sand cats choose to spend the day underground and hunt at night. The way the sand cat hunts is also really fascinating. They may detect prey moving underground because they are highly sensitive to sound frequencies and can hear sounds with low frequencies. The SCSO exhibits notable benefits in terms of enhanced mining capacity and reliable performance in addressing real-world challenges. Nevertheless, the weaknesses of SCSO are obvious. During the sophisticated phase of the SCSO algorithm, the individual sand cat has a tendency to become trapped in a local optima, resulting in lack of ability to identify a more favorable position.

Numerous researchers have conducted research with the objective of strengthening the comprehensive the ability of the SCSO algorithm. The authors Wu et al.^28^ provided a modified approach for tackling limited engineering optimization problems by introducing an improved sand cat swarm optimization problem. Attempting to boost the mobility of sand cats and improve their worldwide discovery ability, the researchers implemented the modified sand cat swarm optimization algorithm (MSCSO) with a loitering strategy. In an attempt to improve the overall performance of the algorithm and expedite convergence, a shot-based reverse learning method is incorporated. A power transformer defect diagnosis approach was proposed by Lu et al.^29^, which utilizes an improved sand Cat swarm optimization algorithm and a unit with a bidirectional gated cycle. The conventional sand Cat swarm algorithm was enhanced through the incorporation of logical chaotic mapping, a water wave dynamic component, adaptive weighting, and a gold sine approach. The superiority of ISCSO in terms of optimization precision and quickness of convergence has been demonstrated. As a result, a fault diagnosis technique utilizing L-Isomap and ISSO-BigRU has been created. The adaptive sand cat swarm optimization algorithm (COSCSO) was proposed by Wang et al.^30^. This approach is based on Nonlinear adaptive parameters、the Cauchy variation and the optimal neighborhood perturbation strategy. The enhanced algorithm has the capability of minimizing the existence of local optima within the population, expediting the rate of convergence, promoting the efficiency of search, and promoting population biodiversity. In their study, Jia et al.^31^ introduced a Sand Cat swarm optimization algorithm that incorporates quasi-reverse learning strategies. This algorithm effectively converts the three-dimensional path planning problem into an objective function derived from a mathematical model. Consequently, the algorithm facilitates the identification of the optimal path while considering the security constraint. Empirical evidence demonstrates that the enhanced algorithm can effectively identify the most advantageous route in various obstacle-laden conditions. The reactive power optimization approach for storage-distribution networks with wind and wind energy was suggested by Shang et al.^32^. The approach incorporates the multi-objective MOSCSO algorithm. The control variable in this research is the energy storage facility, and the simulation experiment runs using MOSCSO. In the exploration and development stages, Jiang et al.^33^ introduced the Cauchy mutation mechanism and Gaussian mutation mechanism, respectively. They additionally created the ISCSO algorithm and enhanced the engine failure detection technique of SDAE using the improved algorithm. The usefulness of the proposed strategy in enhancing average diagnostic accuracy while minimizing average time has been verified. The previously mentioned investigators primarily concentrate on developing the sand cat swarm optimization algorithm through adaptive weights and local variation. These tactics not only strengthen the algorithm's efficiency in various ways but also have potential applications in engineering.

Given the restrictions of conventional SCSO, along with the enhanced approaches put out by other researchers, this study introduces a novel technique called CWXSCSO, which combines elite decentralization and crossbar Sand Cat swarm optimization. The paper's fundamental framework is laid out as follows: The initial portion presents the fundamental optimization process of SCSO. The subsequent section introduces an original dynamic exponential element and implements elite decentralization during the developmental phase. Simultaneously, the algorithm incorporates the crossbar approach to disrupt the previous optimal solution. In the third portion, an aggregate of 15 benchmark test functions is employed to evaluate and compare different improvement strategies. The optimized performance of each strategy is assessed and compared. In Sect. “Comparison between CWXSCSO and different swarm intelligence algorithms”, the efficacy of the CWXSCSO approach is assessed using the identical set of CEC2019 test functions and CEC2021 test functions. This evaluation involves assessing several performance metrics such as the ideal value, median value, standard deviation, convergence curve, and the results of the Wilcoxon rank sum test. Section “Engineering application” of the study employs six classical engineering cases to evaluate the viability of the new algorithm in real-world engineering scenarios. To summarize, when comparing the conventional SCSO algorithm with CWXSCSO, it can be observed that CWXSCSO exhibits a certain degree of effectiveness in extinguishing the algorithm from local optima. Additionally, CWXSCSO strengthens the pace of convergence and solution accuracy of SCSO.

## Sand cat swarm optimization algorithm

The optimization algorithms of sand cat swarms draw inspiration from their capacity to identify low-frequency noise. Sand cats inhabit challenging habitats characterized by sandy and stony deserts, such as the Sahara in Central Asia, the Sahara in Africa, and the Arabian Peninsula. They engage in daily relaxation and nocturnal hunting activities. They participate in prey detection by sensing low-frequency sounds, whether it is above or below ground. When prey is subterranean, they promptly identify it and excavate it. Based on the behavioral patterns exhibited by sand cats, the process of foraging can be delineated into two distinct phases: prey detection and prey predation. The SCSO algorithm places emphasis on two distinct phases, namely exploration and development, with a particular focus on maintaining a balance between these phases.

### Initialize

The sand cat in a D-dimensional optimization problem is a one-dimensional array that operates as a representation of the solution to the problem. Every variable value (${x}_{1}$,${x}_{1}$, …, ${x}_{n}$) represents a node. Additionally, every $X$ must be positioned within the limits of the upper and lower limits.

Initially, an initialization matrix is generated based on the issue size, denoted as $\left({N}_{pop}\times {N}_{d}\right),\left(pop=1,...,n\right)$. Furthermore, the solution that corresponds to the given input is generated in every iteration. If the subsequent output value exhibits higher efficiency, the current approach will be substituted. The solution for the next iteration is not stored if a superior solution is not obtained. Each sand cat's fitness value was established via the fitness function.

$${SandCat}_{i}=\left\{{SC}_{1},{SC}_{2},\dots ,{SC}_{n}\right\};1<i\le n$$

$${Act}_{i}^{j}=\left\{{X}_{11},{X}_{12},\dots ,{X}_{nm}\right\};1<i\le n;1<j\le d$$

$$Fitness=f\left(SandCat\right)=f\left({SC}_{1},{SC}_{2},\dots ,{SC}_{n}\right);\forall {x}_{i}$$

### Hunt for prey

The vector $R$ is derived from Eq. (2). The adaptive parameter R enhances the equilibrium between the transition and development of the two phases.

The parameter $\overrightarrow{{r}_{G}}$ defines a general sensitivity that exhibits a linear reduction from 2 to 0. Furthermore, the variable $r$ symbolizes the sensitivity span exhibited by each cat.

The expression ${\text{ite}}{\text{r}}_{c}$ denotes the present proportion of iterations. The notation ${\text{ite}}{\text{r}}_{M{\text{ax}}}$ denotes the upper limit of iterations. The ${S}_{M}$ number is derived from the acoustic attributes of the sand cat, hence its assumed value is 2.

1 $$\begin{array}{c}{r}_{G}={s}_{M}-\left(\frac{{S}_{M}*{\text{ite}}{\text{r}}_{c}}{{\text{ite}}{\text{r}}_{M{\text{ax}}}}\right)\end{array}$$

2 $$\begin{array}{c}R=2*{r}_{G}*{\text{r}}{\text{a}}{\text{n}}{\text{d}}\left(\mathrm{0,1}\right)-{r}_{G}\end{array}$$

3 $$\begin{array}{c}r={r}_{G}*rand\left(\mathrm{0,1}\right)\end{array}$$

Every individual sand cat adjusts its location based on its most ideal position $\left({X}_{b}\right)$, present position $\left({X}_{c}\right)$, and its sensitivity range $(r)$. Hence, the sand cat possesses the capability to identify alternative ideal prey areas, as determined by Eq. (4).

4 $$\begin{array}{c}X\left(t+1\right)=r*\left({X}_{b}\left(t\right)-{\text{rand}}\left(\mathrm{0,1}\right)*{X}_{c}\left(t\right)\right)\end{array}$$

### Attack prey

The formula (5) denotes the gap ${X}_{rnd}$ between the sand cat and the prey, representing the simulation of the sand cat's attack on the target. Assuming a circular sensitivity span for the sand cat, a position is produced randomly from the best position $\left({X}_{b}\right)$ and the present position $\left({X}_{c}\right)$. Subsequently, a random angle is picked using the roulette method, and the assault is executed using formula (6). The utilization of randomly generated angles can effectively mitigate the risk of the algorithm succumbing to local optima.

5 $$\begin{array}{c}{X}_{rnd}=\left|{\text{rand}}\left(\mathrm{0,1}\right)*{X}_{b}\left(t\right)-{X}_{c}\left(t\right)\right|\end{array}$$

6 $$\begin{array}{c}X\left(t+1\right)={X}_{b}\left(t\right)-r*{X}_{rnd}*\mathit{cos}\left(\theta \right)\end{array}$$

### Exploration and development

The utilization of adaptive values for the $\overrightarrow{{r}_{G}}$ and $R$ parameters facilitates the process of exploration and development, enabling the SCSO to smoothly transition between the two stages. The values of the R argument are considered to be well balanced when the contents of the $\overrightarrow{{r}_{G}}$ argument are spread out in an equitable manner. In a nutshell, the R value is a stochastic value within the range [− 2 $\overrightarrow{{r}_{G}}$, 2 $\overrightarrow{{r}_{G}}$], where $\overrightarrow{{r}_{G}}$ is reduced from 2 to 0 in each iteration. Consequently, the parameter R is a randomized value within the bounds Report Phrase of [− 4, 4].

If the quantity of |R| is less than 1, the sand cats are directed to engage in prey assault. Conversely, if |R| is greater than 1, the cats are assigned the responsibility of identifying a novel potential solution over the entire region.

7 $$\begin{array}{l}X\left(t+1\right)=\left\{\begin{array}{c}r*\left({X}_{b}\left(t\right)-rand\left(\mathrm{0,1}\right)*{X}_{c}\left(t\right)\right);\left|R\right|>1;\,exploration\\ {X}_{b}\left(t\right)-{X}_{rnd}*{\text{cos}}\alpha *r;\left|R\right|\le 1;\,exploitation\end{array}\right.\end{array}$$

## Improved sand cat swarm optimization algorithm

This study presents novel approaches to address the issues of slow convergence and susceptibility to local optima in the SCSO algorithm. Specifically, it introduces a dynamic exponential factor, an elite decentralization technique, and a crossbar strategy as potential enhancements to the SCSO algorithm. The subsequent part contains a detailed introduction to three improvement strategies.

### Dynamic exponential factor

The parameter of the weight factor holds significant importance. With a relatively high weight factor, the algorithm exhibits robust global search capabilities, enabling it to enhance population variety and cover a vast area. When the comparison is small, the algorithm has a robust local search capability, enabling it to efficiently explore the ideal solution and expedite convergence. Local optimization is a process in which the sand cat participates in local search, as described by formula (4). According to formula (6), as the sand cat swarm approaches the local solution, it is restricted to approaching the solution that is deemed to be locally optimal and lacks the ability to achieve superior local optimization. A novel dynamic exponential factor is suggested as a solution to this challenge, drawing inspiration from existing work. This factor can compensate for the limited capacity for local exploitation during the initial phase and improve the overall search capability during the latter phase, so preventing the population from prematurely settling in the local optimal. Equation (8) illustrates an equation for the exponential factor.

8 $$\begin{array}{c}\omega ={\left({e}^{\left(1-{\left(\frac{t}{T}\right)}^{2}\right)}\right)}^{kt}\end{array}$$

In the given context, $k$ represents an optimization factor that adheres to an exponential distribution. The variable $t$ depicts the present quantity of iterations, while $T$ indicates the ultimate amount of iterations.

### Elite decentralization strategy

In the desert, the sand cat's power to update its individual location is limited to relying on the guidance of random individuals within the community, resulting in a weak global search capability. This study shows a novel approach to elite decentralization, aiming toward improving the proximity of individual sand cats to elite individuals and bolstering the local development capabilities of the sand cat population. The ultimate goal is to expedite and improve the sand cat population's ability to identify optimal solutions with greater speed and accuracy.

The calculation of fitness for each individual sand cat is performed, followed by the replication of the individual with the highest level of fitness into n copies, so building the elite matrix. When the probability of |R| is equal to 1, various probabilities h are introduced to update the location. Specifically, when h is equal to or less than 1/3, the population is searched worldwide. This approach addresses the limitation of the original algorithm, which is vulnerable to local optima, and prevents the sand cat population from evolving into "precocious". When the value of h exceeds 0.5, the elite matrix is included, ensuring that each dimension of the sand cat individual is in close alignment to the elite individual. This allows for the rapid identification of the best value.

Please revise formula (6) to update its position to the following formula:

When $$h\le 1/3$$:

9 $$\begin{array}{c}X\left(t+1\right)={\omega *X}_{b}\left(t\right)-{C}_{1}*{\text{cos}}\left({C}_{2}\right)\left|r*{X}_{rnd}*\mathit{cos}\left(\theta \right)\right|+{X}_{E}\end{array}$$

When $$h>1/3$$:

10 $$\begin{array}{c}X\left(t+1\right)=\omega *\left|{X}_{b}\left(t\right)-X\left(t\right)\right|+{X}_{E}\end{array}$$

among:

11 $$\begin{array}{c}{C}_{1}=2*\pi *rand\left(\mathrm{0,1}\right)\end{array}$$

12 $$\begin{array}{c}{C}_{2}=2*\left(1-\frac{t}{T}\right)*{\text{cos}}{\left(\frac{3*\pi *t}{2}\right)}^{3}\end{array}$$

### Crossbar strategy

With a boost in the total amount of iterations, the sand cats within the population tend to cluster around the optimal individuals. This may give rise to the phenomenon of population diversity decline and hinder the algorithm's ability to develop the global optimal solution. This paper incorporates the horizontal crossover strategy into the sand cat swarm optimization algorithm to mitigate the occurrence of local optima within the algorithm. The horizontal crossover is utilized to cross-search the population, thus minimizing search blind spots and addressing the global optimization problem. The vertical crossover operation is executed on the optimal solution for the purpose of addressing early convergence of the algorithm, hence enabling the algorithm to transcend the local optima and enhance the population's variety. The Crossbar technique, as described in reference^34^, has the potential to improve the worldwide search functionality for addressing intricate optimization issues. Consequently, this can result in better precision in solving the algorithm and accelerated convergence speed.

#### Transverse crossing

Horizontal crossover refers to the process of conducting crossover operations across all dimensions of a population for the purpose to facilitate reciprocal learning among distinct individuals. Prior to the implementation of the horizontal crossing approach, all participants of the sand cat population are randomly paired together without any repetition. Subsequently, an arithmetic crossover is conducted, with the probability ${P}_{h}$ typically assigned an estimate of 1. The offspring are formed through the process of crossing the parent generation, as indicated by formula (13), (14):

13 $$\begin{array}{c}{M}_{i,d}^{hc}={q}_{1}*X\left(i,d\right)+\left(1-{q}_{1}\right)*X\left(j,d\right)+{c}_{1}*\left(X\left(i,d\right)-X\left(j,d\right)\right)\end{array}$$

14 $$\begin{array}{c}{M}_{j,d}^{hc}={q}_{2}*X\left(j,d\right)+\left(1-{q}_{2}\right)*X\left(i,d\right)+{c}_{2}*\left(X\left(j,d\right)-X\left(i,d\right)\right)\end{array}$$

where: ${q}_{1}$ and ${q}_{2}$ are random numbers [0,1]; Both ${c}_{1}$ and ${c}_{2}$ are random numbers [-1, 1]. $X\left(i,d\right)$ and $X\left(j,d\right)$ are the parents of d dimension $X\left(i\right)$ and $X\left(j\right)$ respectively. ${M}_{i,d}^{hc}$ and ${M}_{j,d}^{hc}$ represent the D-dimensional progeny of $X\left(i,d\right)$ and $X\left(j,d\right)$ by horizontal crossing, respectively. The generated offspring compete with their parents to retain the best fitness individual.

#### Longitudinal crossing

The SCSO algorithm exhibits a tendency to encounter local optima in subsequent iterations, a phenomenon frequently attributed to the occurrence of local optima in specific dimensions during the updating procedure. The vertical crossover is a type of arithmetic crossover that is applied to all individuals between two distinct dimensions. It involves updating only a specific dimension, so facilitating the evasion of a dimension that is imprisoned in a local optima. The vertical intersection of the ${d}_{1}$ and ${d}_{2}$ dimensions of individual $i$ yields the descendant individuals, as calculated via Eq. (15).

15 $$\begin{array}{c}{M}_{i,{d}_{1}}^{vc}=q*X\left(i,{d}_{1}\right)+\left(1-q\right)*X\left(j,{d}_{2}\right)\end{array}$$

where: $q$ is the random number on [0,1]: ${M}_{i,{d}_{1}}^{vc}$ is the child of parent $X\left(i\right)$ generated by vertical crossing in ${d}_{1}$ and ${d}_{2}$ dimensions. The offspring individuals produced by longitudinal crossing compete with their parents to retain the individuals with better fitness.

### Implementation of CWXSCSO algorithm

The modified sand cat swarm optimization technique suggested in this research follows the basic flow based on the aforementioned enhancement methods.

**Step 1** Establish the initial position of the population and ascertain the parameters;

**Step 2** The objective is to determine the fitness of a sand cat colony by calculating the current ideal location and target value.

**Step 3** The position update formula of the basic SCSO algorithm (4) is utilized to update the current position of the sand cat when the value of |R| is greater than 1.

When the quantity of $R$ is less than 1, the process of choosing the hunting approach is selected based on a random probability $h$. When $h$ is less than or equal to 1/3, the spatial arrangement of the sand cat is updated using the improved position formula (9). Conversely, when $h$ is greater than or equal to 1/3, the spot of the sand cat is updated using the location formula (10).

**Step 4** The horizontal cross operation is performed to cross all dimensions, and the position is updated based on Eqs. (13) and (14).

**Step 5** Based on Formula (15), the longitudinal crossover operation is executed, followed by a comparison of fitness standards, and ultimately, the ideal reserve is selected.

**Step 6** Assess the extent to which the algorithm satisfies the stop condition. If affirmative, exit the primary loop and display the desired location and value; else, revert back to Step 3.

**Step 7** Terminate the program and display the optimal outcome.

### Improved pseudocode of sand cat group optimization algorithm

**Algorithm 1:**
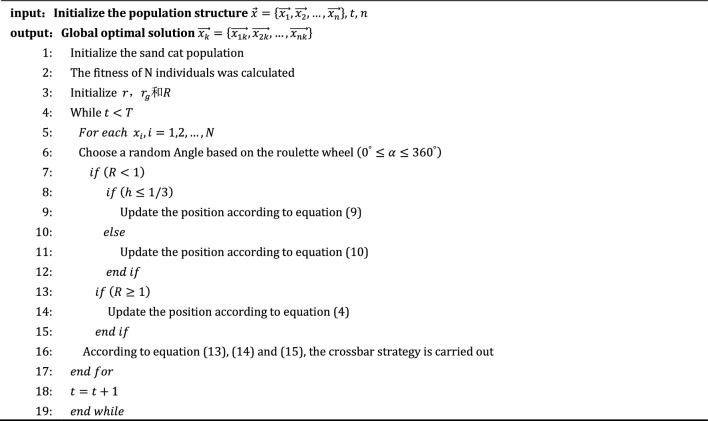
Improved the pseudocode of sand cat swarm optimization algorithm.

### CWXSCSO flow chart

**Figure 1:**
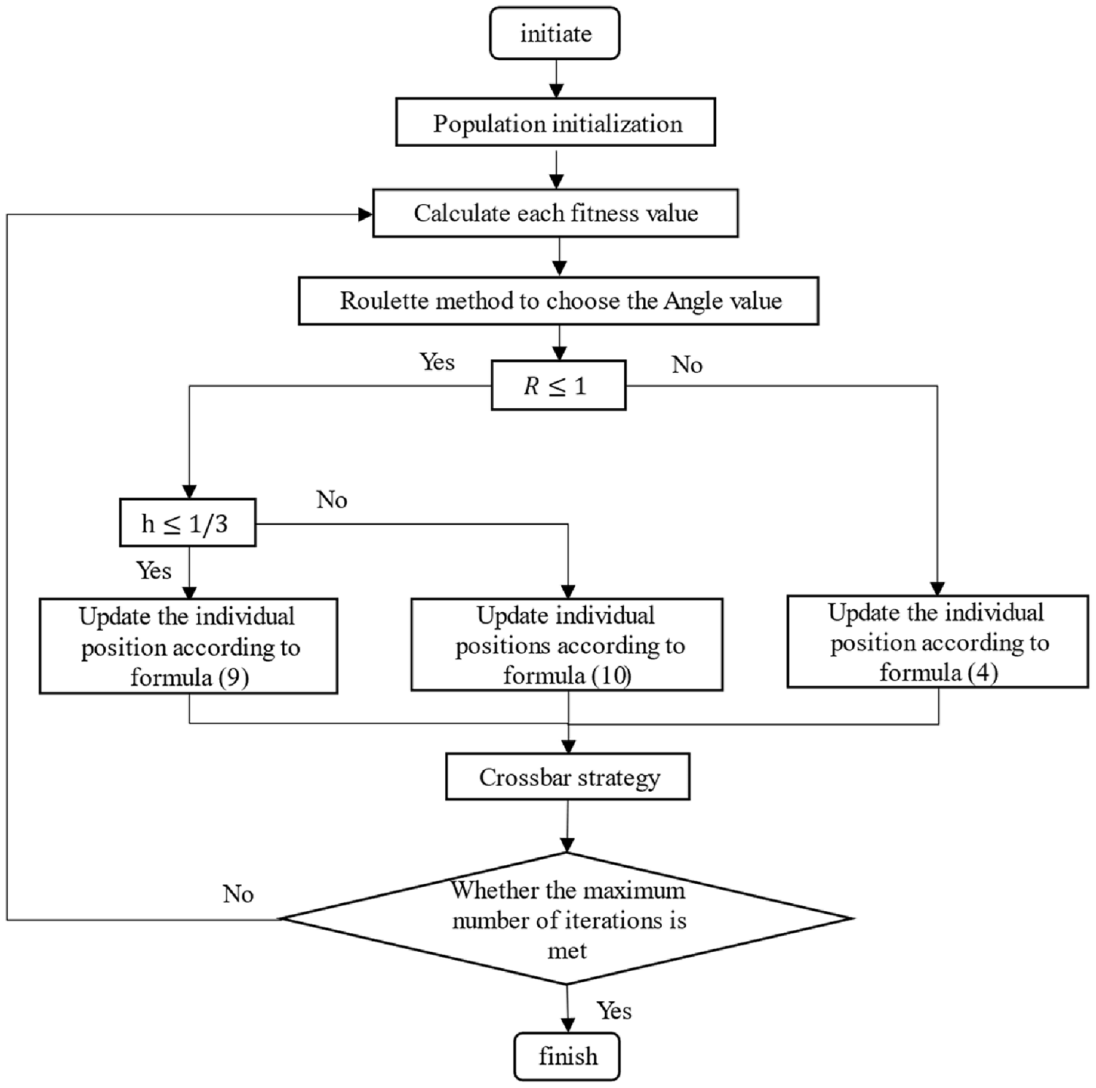
CWXSCSO flow chart.

Figure 1 describes the flow chart of the improved algorithm in detail, as shown below:

### Computational complexity

The numerical representation of the time complexity of an algorithm is commonly denoted as $O$. The CWXSCSO algorithm primarily comprises the dynamic exponential factor, elite decentralization approach, and crossbar strategy. The dimension of the search space is given as $m$ when the population size is $N$, and the greatest amount of iterations is $T$, the time complexity analysis of the algorithm in this paper is as follows: The time complexity of initialization is $O(m)$; The time complexity of calculating the fitness value is the dynamic exponent $O(N+N\times logN)$. The dynamic index is improved on the basis of the original linear weight, and the time complexity is still $O\left(N\times m\right)$ The elite decentralization approach has a temporal complexity of $O\left(N\times m\right)$. The temporal complexity of the crossbar technique can be expressed as $O\left(N\times m/2+m/2\right)=O(N\times m+m)$, and other calculations are small and negligible. Thus, the CWXSCSO method exhibits an overall computational complexity of $O\left(m+T\times N\left(1+m+{\text{log}}N\right)\right)$, aligning with the computational complexity of the conventional sand cat swarm optimization algorithm.

## Simulation experiment and result analysis

### Benchmark function

To assess the efficacy and enhancement of CWXSCSO, a set of 15 benchmark functions was chosen, as outlined in Appendix A1. The function F1-F7 is unimodal, meaning it has just one global ideal and no local optimal. This characteristic allows for a more accurate evaluation of the algorithm's convergence time and optimization accuracy. The function F8-F13 exhibits multidimensionality and multimodality. Multiple local extreme values are frequently employed to assess the algorithm's performance in preventing local optima and facilitating search worldwide. The fixed-dimensional multimodal functions F14 and F15 are being referred to. Multimodal functions exhibit numerous local extrema.

### Experimental results and analysis of reference function

To enhance the verification of the efficacy of each enhanced method in CWXSCSO, three strategies are examined individually. The concept can be categorized into three distinct strategies: dynamic factor (JSCSO), elite decentralization strategy (ZSCSO), and Crossbar strategy (XSCSO). The length of the query space for F1-F15 is fixed at 30, with a total size of $N=50$. Additionally, the upper limit for the number of iterations, denoted as ${T}_{max}$, is limited to 1000. The optimum value, average value, and standard deviation are obtained by executing each function 30 times.

The statistics shown in Tables 1 and 2 demonstrate that the ideal value, median value, and standard deviation of CWXSCSO exhibit improved performance in comparison with SCSO when considering a dimensionality of 30. CWXSCSO, JSCSO, and ZSCSO algorithms achieve the theoretical best value for every round of the F1-F4 function for simple unimodal functions. In the F5 algorithm, the ideal value and mean value of CWXSCSO exhibit inferior performance compared to XSCSO, while the normal deviation is inferior to that of JSCSO. Nevertheless, CWXSCSO still possesses certain advantages when compared to the basic algorithm. The convergence of the method to the theoretical best value for the complex unimodal function F6 is frequently challenging. However, the inclusion of the crossbar method has resulted in improved optimization accuracy for the algorithm. The modified algorithm exhibits a 15-fold increase in accuracy compared to its original counterpart. The CWXSCSO strategy in F7 has superior performance in terms of ideal value, average value, and variance compared to SCSO and other methodologies. CWXSCSO exhibits the maximum optimization accuracy and the lowest standard deviation for multi-modal functions F8, F12, and F13. In conjunction with CWXSCSO, XSCSO exhibits superior optimization efficacy, hence indicating the advantageous nature of employing the crossbar technique to facilitate the algorithm's departure from local optima. All strategies in the F9-F11 function attain the theoretical ideal value, suggesting that the incorporated strategies exhibit favorable stability. Table 2 demonstrates that the optimization accuracy of CWXSCSO is preferable to that of SCSO and other processes for the multi-modal function F14-F15 with fixed dimensions. In simple terms, the revised algorithm incorporates dynamic factors and elite decentralization strategies to enhance its development performance. Additionally, the crossbar technique facilitates the algorithm's ability to transcend local optima.

**Table 1:** The optimization outcomes of the single-peak test function (F1-F6) were compared using various improvement methodologies.

Function	Algorithm	Optimal value	Mean value	Standard deviation
F1	SCSO	1.6505E − 248	1.7794E − 232	0
	JSCSO	0	0	0
	ZSCSO	0	0	0
	XSCSO	6.3662E − 255	4.7234E − 239	0
	CWXSCSO	**0**	**0**	**0**
F2	SCSO	4.9174E − 128	7.4487E − 122	1.6613E − 121
	JSCSO	0	0	0
	ZSCSO	0	0	0
	XSCSO	2.9000E − 132	3.9944E − 127	2.0088E − 126
	CWXSCSO	**0**	**0**	**0**
F3	SCSO	4.6337E − 219	1.2671E − 203	0
	JSCSO	0	0	0
	ZSCSO	0	0	0
	XSCSO	1.0331E − 213	1.2787E − 196	0
	CWXSCSO	**0**	**0**	**0**
F4	SCSO	2.3733E − 110	3.4932E − 103	1.5215E − 102
	JSCSO	0	0	0
	ZSCSO	0	0	0
	XSCSO	3.3619E − 109	4.5124E − 101	2.4371E − 100
	CWXSCSO	**0**	**0**	**0**
F5	SCSO	4.6159E + 01	4.7947E + 01	8.7684E − 01
	JSCSO	4.8102E + 01	4.8839E + 01	**2.1074E** − **01**
	ZSCSO	4.8070E + 01	4.8755E + 01	2.4136E − 01
	XSCSO	**4.4000E + 01**	**4.4501E + 01**	2.5036E − 01
	CWXSCSO	4.5664E + 01	4.6590E + 01	6.9594E − 01
F6	SCSO	1.9686E + 00	3.5213E + 00	7.0735E − 01
	JSCSO	1.0616E + 01	1.1043E + 01	2.4024E − 01
	ZSCSO	9.2714E + 00	1.0675E + 01	4.8528E − 01
	XSCSO	9.9686E − 06	1.6590E − 05	4.0816E − 06
	CWXSCSO	**2.7082E** − **15**	**1.7763E** − **13**	**3.4775E** − **13**
F7	SCSO	9.6579E − 07	4.9265E − 05	5.5692E − 05
	JSCSO	6.5727E − 07	1.8145E − 05	1.4117E − 05
	ZSCSO	1.0667E − 07	1.7231E − 05	1.7336E − 05
	XSCSO	1.1360E − 06	1.4352E − 05	1.4999E − 05
	CWXSCSO	**2.2485E** − **07**	**4.8359E** − **06**	**5.1410E** − **06**

Significant values are in bold.

**Table 2:** The optimization outcomes of multi-peak and fixed-dimension test functions (F8-F15) were compared using various improvement methodologies.

Function	Algorithm	Optimal value	Mean value	Standard deviation
F8	SCSO	− 1.3399E + 04	− 1.1025E + 04	1.0451E + 03
	JSCSO	− 5.0405E + 03	− 3.7293E + 03	4.9570E + 02
	ZSCSO	− 1.1647E + 04	− 9.5069E + 03	1.2672E + 03
	XSCSO	− 2.0930E + 04	2.0477E + 04	2.3510E + 02
	CWXSCSO	** − 2.0949E + 04**	** − 2.0949E + 04**	**9.3256E − 09**
F9	SCSO	0	0	0
	JSCSO	0	0	0
	ZSCSO	0	0	0
	XSCSO	0	0	0
	CWXSCSO	0	0	0
F10	SCSO	4.4409E − 16	4.4409E − 16	
	JSCSO	4.4409E − 16	4.4409E − 16	
	ZSCSO	4.4409E − 16	4.4409E − 16	
	XSCSO	4.4409E − 16	4.4409E − 16	
	CWXSCSO	4.4409E − 16	4.4409E − 16	
F11	SCSO	0	0	0
	JSCSO	0	0	0
	ZSCSO	0	0	0
	XSCSO	0	0	0
	CWXSCSO	0	0	0
F12	SCSO	4.9159E − 02	1.0704E − 01	4.2033E − 02
	JSCSO	8.6098E − 01	9.8243E − 01	7.4996E − 02
	ZSCSO	7.4167E − 01	9.8563E − 01	1.3681E − 01
	XSCSO	3.2517E − 07	5.8252E − 07	1.5196E − 07
	CWXSCSO	**9.7303E − 17**	**2.5354E − 15**	**5.5692E − 15**
F13	SCSO	2.9328E + 00	4.4606E + 00	3.1602E − 01
	JSCSO	4.7350E + 00	4.8159E + 00	3.3593E − 02
	ZSCSO	4.1989E + 00	4.6829E + 00	1.7204E − 01
	XSCSO	1.0363E − 05	1.8981E − 05	4.7085E − 06
	CWXSCSO	**1.5743E − 20**	**1.2104E − 18**	**1.8608E − 18**
F14	SCSO	9.9800E − 01	1.3948E + 00	8.8732E − 01
	JSCSO	2.9821E + 00	4.5383E + 00	3.4798E + 00
	ZSCSO	2.9821E + 00	8.0322E + 00	4.6754E + 00
	XSCSO	9.9800E − 01	9.9800E − 01	1.5260E − 14
	CWXSCSO	**9.9800E − 01**	**9.9800E − 01**	**1.5701E − 16**
F15	SCSO	3.0749E − 04	4.0386E − 04	2.7901E − 04
	JSCSO	4.5646E − 04	7.4476E − 04	1.4641E − 04
	ZSCSO	3.1404E − 04	9.0459E − 04	1.4293E − 03
	XSCSO	3.0749E − 04	3.3801E − 04	1.6718E − 04
	CWXSCSO	**3.0749E − 04**	**3.3062E − 04**	**4.2702E − 05**

Significant values are in bold.

**Figure 2:**
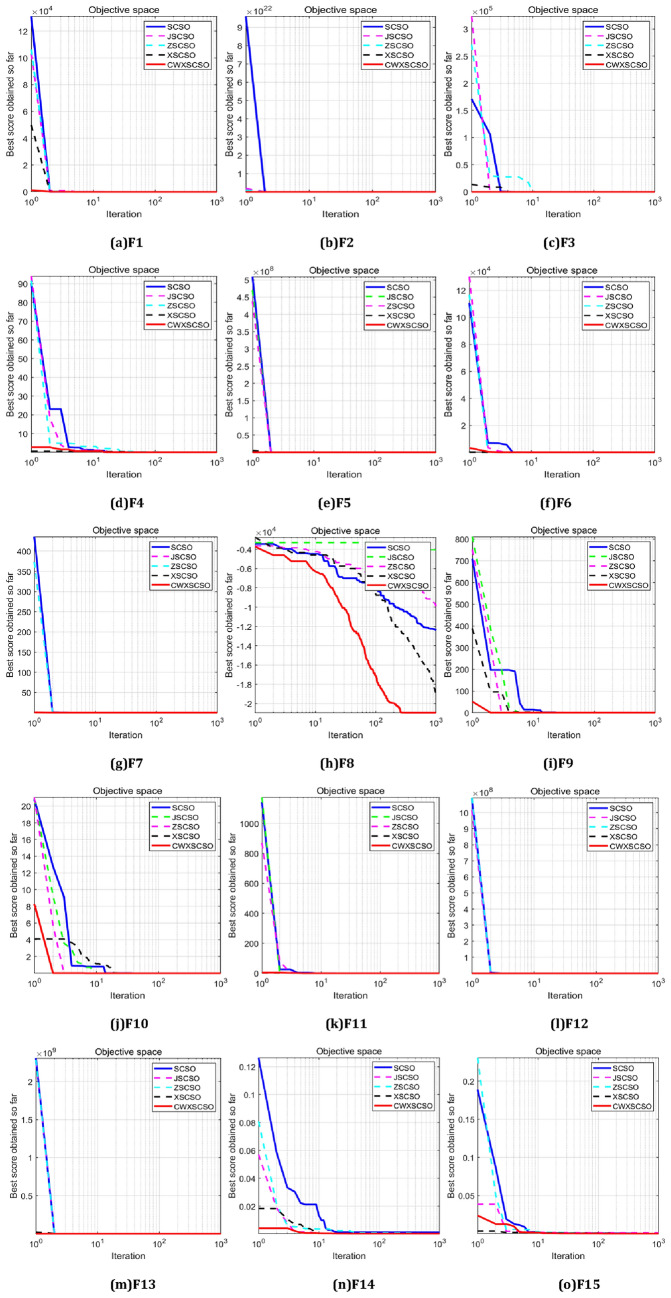
Convergence curve for comparison between strategies.

The iteration rules for functions exhibit a high degree of similarity, as depicted in Fig. 2. The iterative curve of CWXSCSO on the F1-F7 function exhibits a nearly linear pattern, suggesting that the enhanced approach outperforms the original technique with regard to both velocities of convergence and optimization precision. The F8 curve clearly demonstrates that the CWXSCSO algorithm achieved the optimal value after 110 iterations, but the previous algorithm did not reach the optimal value within 1000 iterations. Moreover, the image has multiple inflection points, indicating that the improved algorithm not only has a high fitness value, but also has a good ability to jump out of the local optimal. The curve between F11 and F13 has a linear pattern, suggesting that while the ideal value can be identified, the enhanced method demonstrates superior speed. The convergence speed of F9, F10, F14, and F15 exhibits rapid convergence towards the initial optimal value. Additionally, there are instances where the enhanced algorithm demonstrates the ability to surpass local optima and achieve speedy convergence.

The numerical simulation results, as shown in Tables 1 and 2, along with the convergence diagram depicted in Fig. 2, demonstrate that the CWXSCSO algorithm exhibits superior optimization accuracy, faster convergence acceleration, and better robustness compared to the alternative approach. These findings provide empirical evidence supporting the effectiveness of the algorithm under consideration.

## Comparison between CWXSCSO and different swarm intelligence algorithms

### Test on CEC2019 benchmark function

#### The effectiveness analysis of the improved algorithm

This section presents a selection of six new upgraded algorithms for the purpose of comparing the optimization performance of CWXSCSO with other optimization algorithms. They are Salp swarm algorithm based on craziness and adaptive (CASSA)^35^, Subtraction-Average-Based Optimizer (GSABO)^36^, Grey Wolf Optimization Algorithm Based on Elite Learning for Nonlinear Parameters (IGWO)^37^, Whale optimization algorithm based on chaotic search strategy (CWOA)^38^, Whale Optimization Algorithm Based on Elite Opposition-based and Crisscross Optimization (ECWOA)^34^ and Multi-Strategy Chimp Optimization Algorithm and Its Application of Engineering Problem (EOSMICOA)^39^. The solution to the benchmark function in Appendix A2 has been obtained. The experiment had a population size of $N=50$, with 1000 iterations. Each experiment was completed independently 30 times.

The analysis of Table 3 reveals that the enhanced CWXSCSO algorithm exhibits superior optimization accuracy compared to the other six intelligent optimization algorithms. Furthermore, the improved CWXSCSO algorithm demonstrates greater efficacy on the chosen test functions. The optimization accuracy of CWXSCSO meets the theoretical optimal value of 1 while solving the function F1-F2. Additionally, the average and standard deviations of CWXSCSO are preferable to those of the comparison algorithm. The results produced from the F3, F5, F6, F8, and F10 functions exhibit superior performance compared to the comparison algorithm. The standard deviation of CWXSCSO in function F4 is inferior to that of ECWOA, with just a slight improvement. However, in the F7 function, the ideal value and mean exhibit inferior performance compared to ECWOA, but the standard deviation demonstrates greater accuracy in comparison to ECWOA. In the F9 function, the CWXSCSO algorithm exhibits higher precision comparable to other algorithms. However, it is worth noting that the median value and standard deviation of CWXSCSO are comparatively weaker to those of other algorithms. Hence, it can be ascertained that while certain values of the enhanced algorithm presented in this study may not align with the theoretical ideal value, it generally exhibits superiority over other algorithms and possesses advantages in the realm of function optimization problems. This observation further underscores the efficacy of the proposed method.

**Table 3:** Compare the results with his intelligent algorithm.

		CASSA	GSABO	IGWO	CWOA	ECWOA	EOSMICOA	SCSO	CWXSCSO
F1	Best	1	1	1	1	3474.3991	6260.3105	1	**1**
	Mean	1	1	1	1	10,000.6681	10,000.6681	1	**1**
	Std	0	0	0	0	8518.0766	477,985.0910	0	**0**
F2	Best	1.4091	1.00E + 20	8.5056	4.0767	1.4099	1.4933	2.3151	**1.4091**
	Mean	2.9881	1.00E + 20	9.2246	5.1725	4.5412	2.1452	2.7594	**1.4092**
	Std	2.2330	1.7639	1.0168	1.5497	4.4283	0.9219	0.6284	**1.53E − 05**
F3	Best	1.1230	11.4670	75.4821	25.3538	1.4307	3.2677	1.5556	**1.0207**
	Mean	1.1526	18.5796	96.4431	31.0024	1.4526	4.4711	1.6968	**1.0672**
	Std	0.2248	0.0815	7.6634	20.8279	0.1307	12.1507	1.3174	**0.0715**
F4	Best	2.5058	10.2528	10.2574	9.1796	2.3958	6.4279	6.2361	**2.1589**
	Mean	3.8835	10.3381	10.8924	10.0072	3.9861	7.0716	7.2697	**3.6835**
	Std	1.9484	0.1206	0.8980	1.1704	2.2490	0.9103	1.4617	2.1561
F5	Best	1.2141	1.1329	77.7605	23.5512	1.2593	3.2219	1.4557	**1.1203**
	Mean	1.3501	1.3484	94.1279	28.1548	1.4679	3.9688	1.6677	**1.2474**
	Std	0.2108	0.2175	14.1900	4.4841	0.2427	1.0345	0.2204	**0.1262**
F6	Best	3.1971	3.3326	5.0071	4.1673	3.4876	3.8973	3.2628	**3.0976**
	Mean	3.9949	4.0924	5.2635	4.6559	3.9699	4.5614	4.0700	**3.7678**
	Std	0.5302	0.4634	0.3457	0.3097	0.3837	0.4100	0.7014	**0.2903**
F7	Best	1.1795	3.3630	3.8032	1.4946	**1.1436**	1.2602	1.2684	1.2625
	Mean	1.3022	3.4388	3.9093	1.5758	**1.2892**	1.2708	1.3057	1.3136
	Std	0.1735	0.1071	0.1500	0.1149	0.2058	**0.0150**	0.0528	0.0723
F8	Best	21	21.3679	21.5129	21.3529	21.0038	21.3571	21.0295	**21**
	Mean	21.0483	21.3932	21.5931	21.4019	21.0045	21.4181	21.0904	**21.0002**
	Std	0.0683	0.0358	0.1134	0.0694	9.58E − 04	0.0862	0.0861	**2.90E − 04**
F9	Best	1.1473	1.2329	2.9577	1.5190	1.1462	1.1723	1.2199	**1.1180**
	Mean	**1.2021**	1.3221	3.4002	1.5839	1.3918	1.2529	1.3176	1.3344
	Std	0.0955	0.1036	0.3092	**0.0593**	0.2111	0.0625	0.0751	0.1853
F10	Best	20.9196	20.9814	21.3684	21.3097	21.0005	21.3421	21.0485	**20.9039**
	Mean	21.0430	21.0153	21.4347	21.3790	21.0073	21.4387	21.0491	**20.9908**
	Std	0.1121	0.0556	0.0501	0.0672	0.0091	0.0909	0.0982	**0.0085**

Significant values are in bold.

**Figure 3:**
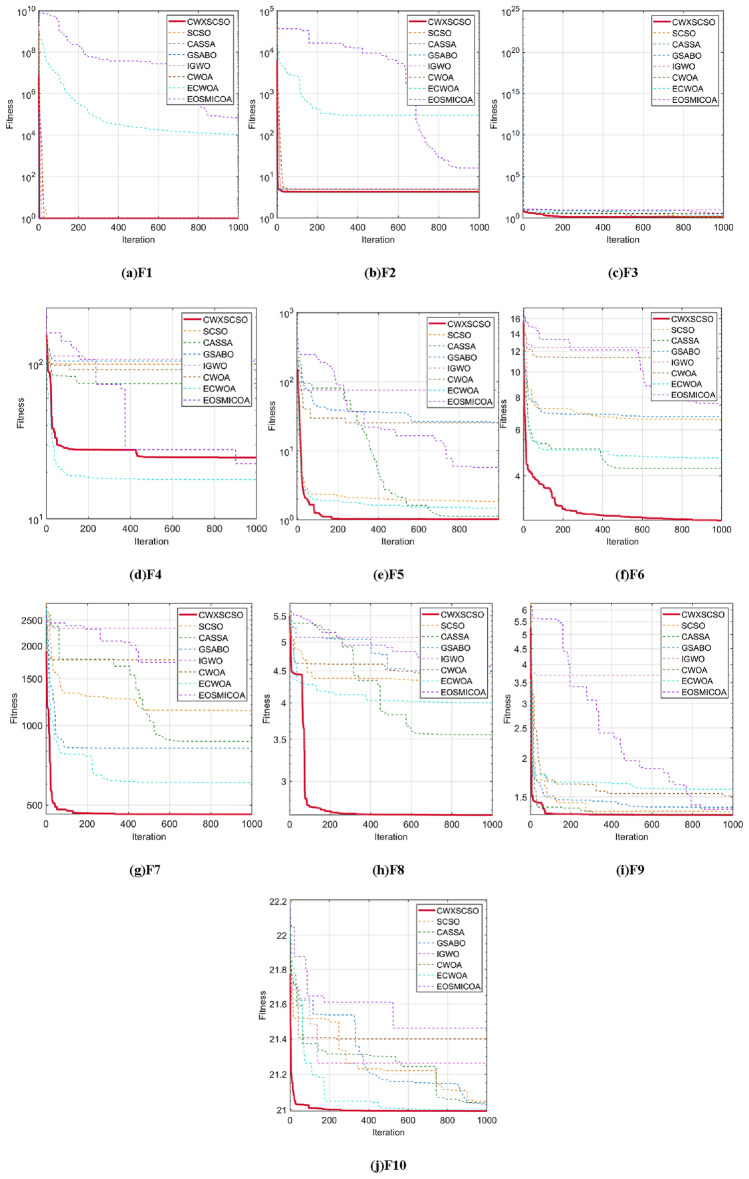
The fitness curves of each algorithm are compared.

The convergence properties of CWXSCSO can be easily observed in Fig. 3, in comparison to other methods. The figure clearly demonstrates that in functions F1 and F2, the curve exhibits rapid convergence and can swiftly approach the global optimal value. In function F3, the population rapidly attains an ideal value, which exhibits superior accuracy compared to alternative algorithms. Despite the relatively low solution accuracy in the F4 and F5 images, the function nevertheless offers several advantages beyond the original approach and other techniques. The optimization of F6-F10 exhibits several nuanced inflection points, suggesting that the algorithm demonstrates an exceptional capacity to overcome local optima and achieve stronger convergence accuracy as opposed to other techniques. In a nutshell judging on the CEC2019 test, CWXSCSO outperforms other algorithms.

#### Wilcoxon rank sum test

The Wilcoxon rank sum test is a non-parametric statistical test that can be utilized irrespective of the distribution of the subject under investigation and the availability of information regarding the distribution. Hence, it is frequently employed to assess the data distribution of two sets of autonomous samples that deviate from a normal distribution. This approach uses the rank of the sample as a substitute for the sample value in order to facilitate data comparison, thereby mitigating the impact of a single value within the sample on the entire sample. Hence, this approach can provide a more scientific representation of the algorithm's optimization performance contrasted to the median value and standard deviation. This section presents a comparison and analysis of the findings obtained from the CEC2019 test. The optimization outcomes of CASSA, GSABO, IGWO, CWOA, ECWOA, and EOSMICOA were compared using the Wilcoxon rank sum test, based on the CWXSCSO method. The hypothesis test employs a significance level of $p=5\%$ as the criterion for judgment. At a significance level of 5%, it can be concluded that there is a substantial difference between the two groups of samples.

In Table 4, the presence of $NaN$ indicates that there lacks a statistically noteworthy disparity between the two groups of data. Additionally, the symbols " + ", " = ", and " − " are used to indicate that the outcomes of the CWXSCSO algorithm outperforms, is equivalent to, or falls short of the comparison algorithm, appropriately. The findings indicate that CWXSCSO exhibits a notable advantage over both the novel method and existing enhanced optimization techniques.

**Table 4:** Results of Wilcoxon rank sum test for CEC2019 functions.

Function	CWXSCSO-CASSA	CWXSCSO-GSABO	CWXSCSO-IGWO	CWXSCSO-CWOA	CWXSCSO-ECWOA	CWXSCSO- EOSMICOA
F1	1.2118e − 12	1.2118e − 12	1.2118e − 12	$NaN$	1.2118e − 12	1.2118e − 12
F2	1.2118e − 12	1.2118e − 12	1.2118e − 12	$NaN$	1.2118e − 12	1.2118e − 12
F3	2.9727e − 2	3.0199e − 11	3.0199e − 11	3.0199e − 11	2.0095e − 1	2.3715e − 10
F4	3.0199e − 11	3.0199e − 11	3.0199e − 11	3.0199e − 11	3.0199e − 11	3.0199e − 11
F5	9.7555e − 10	3.0199e − 11	5.8737e − 4	3.5638e − 4	9.7555e − 10	3.0199e − 11
F6	2.2798e − 11	2.2798e − 11	2.2798e − 11	2.2798e − 11	2.443e − 3	2.2798e − 11
F7	3.0199e − 11	3.0199e − 11	3.0199e − 11	3.0199e − 11	3.0199e − 11	3.0199e − 11
F8	3.0199e − 11	3.0199e − 11	3.0199e − 11	3.0199e − 11	3.0199e − 11	3.0199e − 11
F9	5.4991e − 3	4.5356e − 09	2.5464e − 11	2.5464e − 11	8.5026e − 09	2.5464e − 11
F10	2.7071e − 1	2.9972e − 11	3.3384e − 11	1.5292e − 05	3.0339e − 3	3.0199e − 11
+ / = / −	9/0/1	10/0/0	10/0/0	8/2/0	9/0/1	10/0/0

### Test on CEC2021 benchmark function

#### Validity analysis on CEC2021 function

With the aim to comprehensively evaluate the optimization capabilities of the upgraded method, a set of 10 CEC2021 test functions with distinct optimization features were chosen (as illustrated in Appendix A3). The CWXSCSO is computed and thereafter compared to the previous six enhancements. The parameters are uniformly set to the total size $N=50$, the upper limit number of iterations ${T}_{max}=1000$, and the dimension $d=10$. Each function was simulated 30 times to obtain the optimum value, the average value, and standard deviation of the result. A plot is generated to depict the convergence curves of ten functions.

Based on the analysis of the data presented in Table 5, it turns out apparent CWXSCSO has higher performance relative to other algorithms in terms of both the ideal value and average value over the ten functions. The C3 function exhibits superior minimum and average values compared to GSABO and IGWO, while its standard deviation surpasses that of IGWO and EOSMICOA. This observation indicates that the enhanced algorithm lacks robust flexibility to the C3 function. The lowest and average values of functions C7 and C9 are optimal. However, the standard deviation is suboptimal. The algorithm exhibits a wide variety of fluctuations, although it possesses the capability to get the ideal theoretical value. The CWXSCSO algorithm outperforms other algorithms in functions C1, C2, C4-C6, C8, and C10, exhibiting superior values with a narrow range of fluctuations and excellent precision.

**Table 5:** Compared optimization results of different intelligent algorithms.

		CASSA	GSABO	IGWO	CWOA	ECWOA	EOSMICOA	SCSO	CWXSCSO
C1	Best	1.2770E + 02	1.2993E + 09	7.0927E + 09	1.1002E + 09	1.1900E + 03	1.0502E + 08	2.7518E + 03	**1.2365E + 02**
	Mean	4.4886E + 03	2.5211E + 09	1.0700E + 10	3.8009E + 09	9.4817E + 03	2.6645E + 08	3.6662E + 07	**1.4211E + 03**
	Std	4.0543E + 03	1.1013E + 09	2.2716E + 09	1.8719E + 09	7.5948E + 03	2.0107E + 08	1.1572E + 08	**2.0781E + 03**
C2	Best	1.7667E + 03	2.3346E + 03	3.2079E + 03	2.3391E + 03	1.6261E + 03	2.2988E + 03	1.4653E + 03	**1.4790E + 03**
	Mean	1.9982E + 03	2.4861E + 03	3.3776E + 03	2.5867E + 03	1.7865E + 03	2.4907E + 03	1.9857E + 03	**1.7264E + 03**
	Std	1.6752E + 02	1.5764E + 02	1.5839E + 02	2.2368E + 02	2.1225E + 02	1.4459E + 02	3.7318E + 02	**1.3608E + 02**
C3	Best	7.7204E + 02	7.9987E + 02	7.9485E + 02	**7.2364E + 02**	7.4722E + 02	7.3684E + 02	7.2538E + 02	7.7204E + 02
	Mean	8.1813E + 02	8.2776E + 02	8.0825E + 02	**7.3987E + 02**	7.5915E + 02	7.6649E + 02	7.4936E + 02	8.1813E + 02
	Std	1.8206E + 01	1.4039E + 01	**9.3858E + 00**	1.7625E + 01	1.2128E + 01	2.1164E + 01	2.0668E + 01	1.8206E + 01
C4	Best	2.1189E + 03	2.7864E + 03	2.9433E + 04	2.1943E + 03	2.5204E + 03	2.1458E + 03	1.9325E + 03	**1.9023E + 03**
	Mean	4.3398E + 03	6.0417E + 03	7.4329E + 05	6.1336E + 05	1.1093E + 04	1.3695E + 04	7.5499E + 03	**2.6462E + 03**
	Std	2.4632E + 03	2.5662E + 03	6.9340E + 05	7.1498E + 05	9.6835E + 03	6.9745E + 03	5.7211E + 03	**1.6531E + 03**
C5	Best	6.4462E + 03	3.4701E + 05	7.5719E + 04	2.0987E + 04	6.2107E + 03	2.9823E + 03	2.3850E + 03	**1.7081E + 03**
	Mean	1.8034E + 04	6.8947E + 05	1.6888E + 05	5.7084E + 04	5.7894E + 04	4.4089E + 03	7.4505E + 03	**1.7026E + 03**
	Std	1.1607E + 04	2.0154E + 05	1.0430E + 05	2.7900E + 04	4.2677E + 04	8.3703E + 02	4.0438E + 03	**2.5760E + 00**
C6	Best	1.6069E + 03	1.7253E + 03	2.0057E + 03	1.9540E + 03	1.6012E + 03	1.7437E + 03	1.6206E + 03	**1.6012E + 03**
	Mean	1.8896E + 03	2.0374E + 03	2.3068E + 03	2.0543E + 03	1.7799E + 03	1.8695E + 03	1.7903E + 03	**1.6613E + 03**
	Std	8.1860E + 01	1.7762E + 02	1.4360E + 02	8.1340E + 01	1.4419E + 02	1.0423E + 02	1.6925E + 02	**7.7536E + 01**
C7	Best	6.3967E + 03	3.8404E + 03	1.2623E + 04	6.9373E + 03	3.5546E + 03	5.5198E + 03	2.5484E + 03	**2.5220E + 03**
	Mean	9.9662E + 03	1.5493E + 04	2.3631E + 04	1.1691E + 04	1.0403E + 04	7.7588E + 03	7.5426E + 03	**6.8049E + 03**
	Std	3.5773E + 03	8.6727E + 03	9.2201E + 03	4.7531E + 03	4.7816E + 03	**1.4491E + 03**	5.9916E + 03	3.5373E + 03
C8	Best	2.2247E + 03	2.3359E + 03	2.7625E + 03	2.5119E + 03	2.2358E + 03	3.5616E + 03	2.3015E + 03	**2.2203E + 03**
	Mean	2.2989E + 03	2.5472E + 03	3.0783E + 03	2.6354E + 03	2.3003E + 03	3.8722E + 03	2.3120E + 03	**2.2976E + 03**
	Std	3.3494E + 01	6.1475E + 02	2.1883E + 02	5.1482E + 01	5.3854E + 00	1.4566E + 02	8.1098E + 00	**4.2554E + 00**
C9	Best	2.7391E + 03	2.5728E + 03	2.8215E + 03	2.7478E + 03	2.7469E + 03	2.7703E + 03	2.5007E + 03	**2.5000E + 03**
	Mean	2.7529E + 03	2.7102E + 03	2.9502E + 03	2.8016E + 03	2.7720E + 03	2.7751E + 03	2.6671E + 03	**2.6512E + 03**
	Std	1.8449E + 01	1.3534E + 02	7.9333E + 01	4.8917E + 01	3.2469E + 01	4.4290E + 00	1.2739E + 02	1.3806E + 02
C10	Best	2.8978E + 03	2.9234E + 03	3.2134E + 03	2.9804E + 03	2.8981E + 03	2.9151E + 03	2.9070E + 03	**2.8978E + 03**
	Mean	2.9466E + 03	3.1055E + 03	3.3319E + 03	3.1321E + 03	2.9294E + 03	2.9573E + 03	2.9387E + 03	**2.9317E + 03**
	Std	2.3832E + 01	1.4414E + 02	5.2824E + 01	8.8272E + 01	2.6473E + 01	2.6135E + 01	2.6639E + 01	**2.3378E + 01**

Significant values are in bold.

**Figure 4:**
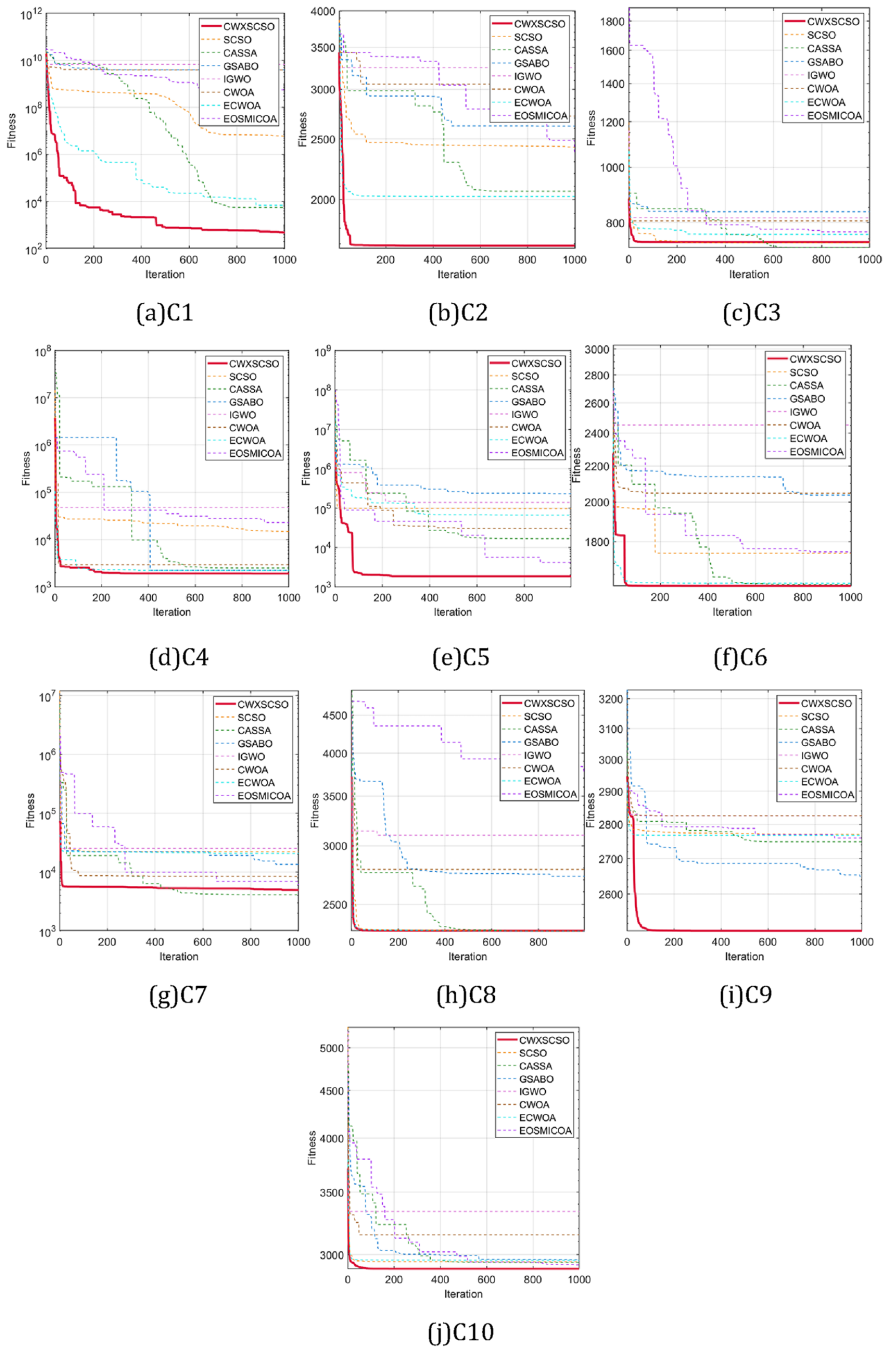
Convergence curves of different algorithms.

Figure 4 clearly demonstrates that all images of functions C1-C10 exhibit significant slopes, suggesting that the enhanced technique achieves faster convergence compared to existing optimization strategies. Photos C1, C4-C6, and C9 exhibit several twists in the CWXSCSO lines, suggesting that the enhanced CWXSCSO possesses the capability to transcend local optima. Contrary to CWXSCSO, other comparison algorithms like EOSMICOA have the capacity to exit the local optimal at numerous iterations. However, their rates of convergence rapidity and precision are not as great. CWXSCSO demonstrates rapid convergence to the global optimal in photos C3, C7, C8, and C10. In images, C3 and C8, the optimal fitness value of CWXSCSO convergence are comparable to that of the original method and CASSA algorithm, but it exhibits the fastest convergence speed.

To summarize, the CEC2021 function test demonstrates that the CWXSCSO algorithm outperforms competing algorithms, hence confirming its effectiveness.

#### Wilcoxon rank sum test of CEC2021 test function

This section presents a comparison and analysis of the findings obtained from the CEC2021 test, with the aim of enhancing the evaluation of the algorithm's optimization performance. The optimization outcomes of CASSA, GSABO, IGWO, CWOA, ECWOA, and EOSMICOA were compared using the Wilcoxon rank sum comparison test, based on the CWXSCSO method. According to the analysis of the results in Table 6, it indicate that CWXSCSO has no significant disparity with the comparison algorithm across several functions. However, none of the functions is inferior to the algorithm, and almost all of the functions demonstrate superior performance in comparison to alternative algorithms. Hence, it may be inferred that CWXSCSO has excellent results compared to both the new method and existing enhanced optimization techniques.

**Table 6:** Results of Wilcoxon rank sum test for CEC2021 test.

Function	CWXSCSO-CASSA	CWXSCSO-GSABO	CWXSCSO-IGWO	CWXSCSO-CWOA	CWXSCSO-ECWOA	CWXSCSO- EOSMICOA
C1	1.2118e − 12	1.2118e − 12	$NaN$	$NaN$	1.2118e − 12	1.2118e − 12
C2	2.9343e − 05	$NaN$	$NaN$	$NaN$	4.7899e − 06	1.3205e − 4
C3	1.9457e − 09	$NaN$	$NaN$	$NaN$	$NaN$	1.6572e − 11
C4	$NaN$	$NaN$	$NaN$	$NaN$	$NaN$	$NaN$
C5	1.2118e − 12	1.2118e − 12	$NaN$	$NaN$	1.2118e − 12	1.2118e − 12
C6	1.2118e − 12	1.2118e − 12	$NaN$	1.7016e − 08	1.2118e − 12	1.2118e − 12
C7	1.2118e − 12	1.2118e − 12	1.608e − 2	3.4526e − 07	1.2118e − 12	1.2118e − 12
C8	1.6082e − 12	$NaN$	$NaN$	$NaN$	$NaN$	$NaN$
C9	1.1364e − 11	2.9785e − 12	2.9009e − 07	7.8379e − 08	3.4571e − 12	1.271e − 11
C10	2.6286e − 11	2.6286e − 11	2.6286e − 11	2.6286e − 11	2.6286e − 11	2.6286e − 11
+ / = / −	9/1/0	6/4/0	3/7/0	4/6/0	7/3/0	8/2/0

## Engineering application

Six engineering challenges have been selected for this part for the purpose to assess how well CWXSCSO performs when used to engineering optimization problems. The sine and cosine optimization algorithm (SCA)^40^, frost and ice optimization algorithm (RIME)^41^, butterfly optimization algorithm (BOA)^42^, Harris Eagle Optimization algorithm (HHO)^8^, and Osprey optimization algorithm (OOA)^43^ were chosen as the primary three technical applications. The whale optimization algorithm (WOA)^7^, the locust optimization algorithm (GOA)^44^, the gray wolf optimization algorithm (GWO)^45^, the marine predator optimization algorithm (MPA)^46^, and the frost and ice optimization algorithm (RIME) were used to compare the final three technical applications. Every algorithm in the experiment has a population of 30 and an upper limit of 1000 iterations.

### Pressure vessel design problems

The performance of the modified algorithm pair gets assessed using pressure vessel design issues in this research. The main objective of the pressure vessel design challenge is to decrease the production expenses associated with the pressure vessel. This problem contains the selection of four optimization variables, namely shell thickness ${T}_{S}$, head thickness (${T}_{h}$), inner radius ($R$), and length of cylinder section without head ($L$). The mathematical description of the pressure vessel design problem is as follows:

variable:

$$\overrightarrow{x}=\left[{x}_{1} {x}_{2} {x}_{3} {x}_{4}\right]=\left[{T}_{S} {T}_{h} R L\right]$$

Function:

$$f\left(\overrightarrow{x}\right)=0.6224{x}_{1}{x}_{3}{x}_{4}+1.7781{x}_{2}{x}_{3}^{2}+3.1661{x}_{1}^{2}{x}_{4}+19.84{x}_{1}^{2}{x}_{3}$$

Constraint condition:

$${g}_{1}\left(\overrightarrow{x}\right)=-{x}_{1}+0.0193{x}_{3}\le 0$$

$${g}_{2}\left(\overrightarrow{x}\right)=-{x}_{3}+0.00954{x}_{3}\le 0$$

$${g}_{3}\left(\overrightarrow{x}\right)=-\pi {x}_{3}^{2}-\frac{4}{3}\pi {x}_{3}^{3}+1296000\le 0$$

$${g}_{4}\left(\overrightarrow{x}\right)={x}_{4}-240\le 0$$

Variable interval:

$$0\le {x}_{1},{x}_{2}\le 99, 10\le {x}_{3},{x}_{4}\le 200$$

**Figure 5:**
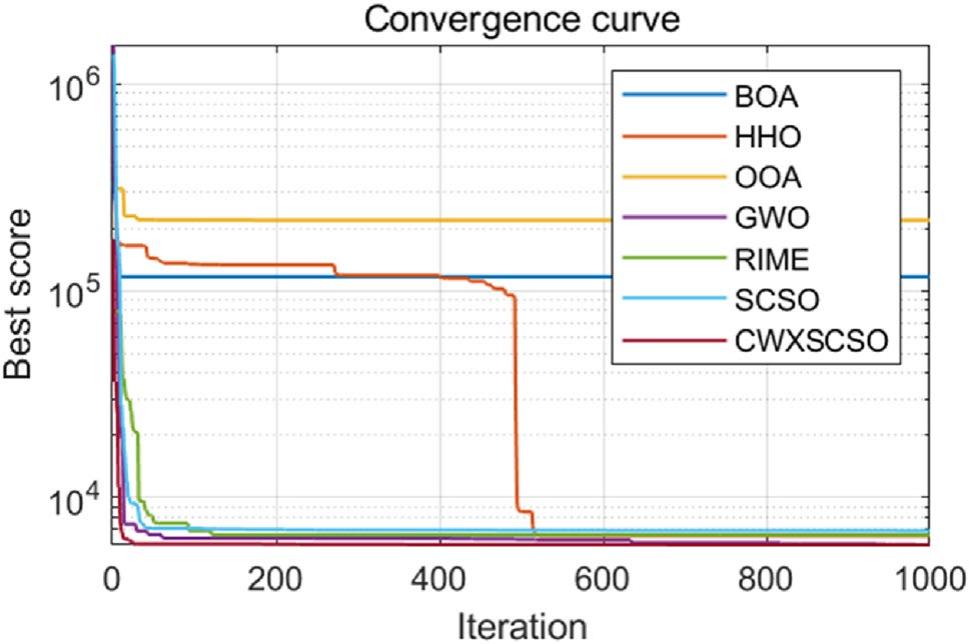
Optimization convergence diagram of pressure vessel design problem.

The experimental findings of CWXSCSO and the comparison algorithm are presented in Table 7. The CWXSCSO yields a value of 5886.05. When compared to alternative algorithms, this particular algorithm exhibits a superior competitive advantage in terms of maintaining the proper functioning of the pressure vessel while simultaneously minimizing costs. Benefits in guaranteeing the operation of the pressure vessel while reducing expenses. The updated method demonstrates rapid convergence to the ideal value with the best convergence accuracy, as depicted in Fig. 5. In turn, the CWXSCSO facility exhibits exceptional engineering optimization capabilities.

**Table 7:** Optimization results of pressure vessel design problems.

Algorithm	${T}_{S}\left({x}_{1}\right)$	${T}_{h}\left({x}_{2}\right)$	$R\left({x}_{3}\right)$	$L\left({x}_{4}\right)$	Result
BOA	1.7058	2.1120	63.6295	37.6427	21,767.48
HHO	1.0975	0.5495	56.2523	55.3661	6774.49
OOA	4.4124	13.0999	54.7594	64.5801	104,691.28
SCA	0.7830	0.3883	40.5700	196.6224	5899.53
RIME	1.2599	0.6233	65.2280	10.0000	7331.08
SCSO	0.8063	0.3986	41.7742	180.7003	5936.00
CWXSCSO	0.7783	0.3847	40.3262	199.9175	5886.05

### Welding beam design problems

The issue at hand is the Welded Beam Design (WBD), which involves the utilization of an optimization method to minimize the production cost associated with the design. The optimization problem can be boiled down to the identification of four design variables that meet the constraints of shear stress $(\tau )$, bending stress $(\theta )$, beam bending load $\left({P}_{c}\right)$, end deviation $(\delta )$, and boundary conditions, namely beam length $(l)$, height $(t)$, thickness $(b)$, and weld thickness $(h)$. The objective is to minimize the manufacturing cost of welded beams. The problem of welded beams is a common example of a nonlinear programming problem. The mathematical description of the welded beam design problem is as follows:

Variable:

$$\overrightarrow{x}=\left[{x}_{1} {x}_{2} {x}_{3} {x}_{4}\right]=\left[h l t b\right]$$

Function:

$$f\left(\overrightarrow{x}\right)=1.10471{x}_{1}^{2}{x}_{2}+0.04811{x}_{3}{x}_{4}\left(14.0+{x}_{2}\right)$$

Constraint condition:

$${g}_{1}\left(\overrightarrow{x}\right)=\tau \left(\overrightarrow{x}\right)-{\tau }_{max}\le 0$$

$${g}_{2}\left(\overrightarrow{x}\right)=\sigma \left(\overrightarrow{x}\right)-{\sigma }_{max}\le 0$$

$${g}_{3}\left(\overrightarrow{x}\right)=\delta \left(\overrightarrow{x}\right)-{\delta }_{max}\le 0$$

$${g}_{4}\left(\overrightarrow{x}\right)={x}_{1}-{x}_{4}\le 0$$

$${g}_{5}\left(\overrightarrow{x}\right)=P-{P}_{c}\left(\overrightarrow{x}\right)\le 0$$

$${g}_{6}\left(\overrightarrow{x}\right)=0.125-{x}_{1}\le 0$$

$${g}_{7}\left(\overrightarrow{x}\right)=1.10471{x}_{1}^{2}{x}_{2}+0.04811{x}_{3}{x}_{4}\left(14.0+{x}_{2}\right)-5.0\le 0$$

Variable interval:

$$0.1\le {x}_{1}\le 2,{ 0.1\le x}_{2}\le 10, 0.1\le {x}_{3}\le 10 ,{0.1\le x}_{4}\le 2$$

**Figure 6:**
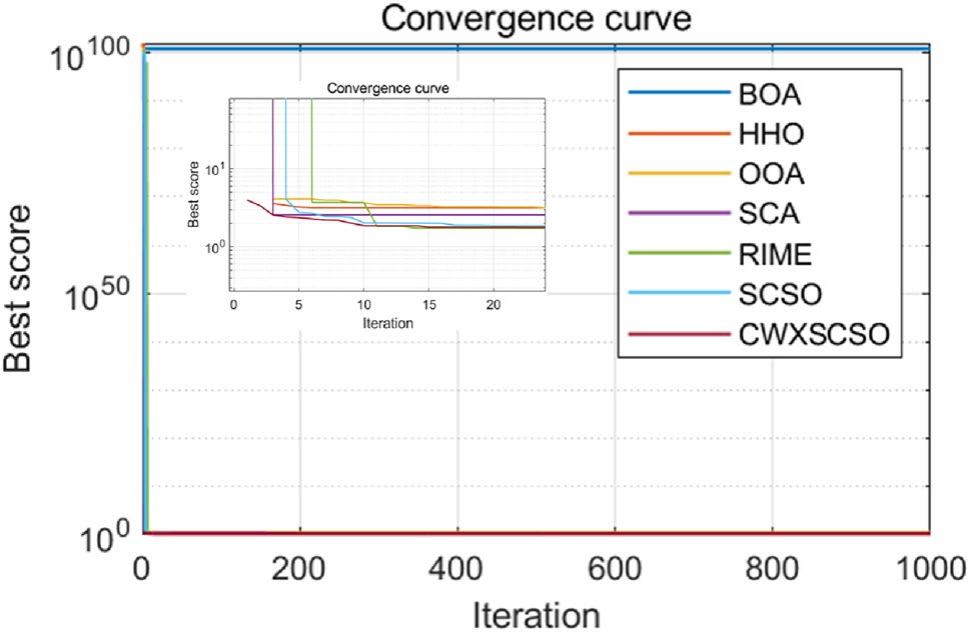
Optimization convergence diagram of welding beam design problem.

As can be seen from Table 8, the final result of CWXSCSO is 1.6935. As can be seen in Fig. 6, the initial fitness value of the improved algorithm is already very good, and there are several subtle turns later, indicating that it has the ability to jump out of the local optimal. The improved algorithm achieves the purpose of reducing the manufacturing cost, and the cost of manufacturing welded beams is minimal compared with other algorithms.

**Table 8:** Optimization results of welding beam design problems.

Algorithm	$h$	$l$	$t$	$b$	Result
BOA	0.1682	8.0508	8.8515	0.2168	2.2875
HHO	0.2087	3.1115	9.1852	0.2113	1.7476
OOA	0.7460	2.4806	4.7436	0.7467	4.3336
SCA	0.2071	3.1690	9.2701	0.2095	1.7547
RIME	0.2157	3.5682	7.9583	0.2654	1.9687
SCSO	0.2038	3.2750	9.0367	0.2057	1.6954
CWXSCSO	0.2058	3.2359	9.0342	0.2058	1.6935

### Reducer design problem

The reducer holds an important place within mechanical systems as a crucial component of the gear box, serving a diverse range of applications. The primary aim of this challenge is to diminish the overall weight of the reducer through the optimization of the seven parameter variables. They are the tooth surface width $b$(= ${x}_{1}$), the gear module $m(={x}_{2})$, the tooth count in the pinion $z(={x}_{3})$, the measurement of the initial shaft distance between bearings. ${l}_{1}(={x}_{4})$, the distance between the bearings of the second shaft ${l}_{2}(={x}_{5})$, the diameter of the initial shaft ${d}_{1}(={x}_{6})$ and the measurement of the diameter of the second shaft ${d}_{2}(={x}_{7})$. The mathematical description of the speed reducer design problem is as follows:

Variable:

$$\overrightarrow{x}=\left[{x}_{1} {x}_{2} {x}_{3} {x}_{4} {x}_{5} {x}_{6} {x}_{7}\right]=\left[b m z {l}_{1} {l}_{2} {d}_{1} {d}_{2}\right]$$

Function:

$$f\left(\overrightarrow{x}\right)=0.7854{x}_{1}{x}_{2}^{2}\left(3.3333{x}_{3}^{2}+14.9334{x}_{3}-43.0934\right)-1.508{x}_{1}\left({x}_{6}^{2}+{x}_{7}^{2}\right)+7.4777\left({x}_{6}^{3}+{x}_{7}^{3}\right)+0.7854({x}_{4}{x}_{6}^{2}+{{x}_{5}x}_{7}^{2})$$

Constraint condition:

$${g}_{1}\left(\overrightarrow{x}\right)=\frac{27}{{x}_{1}{x}_{2}^{2}{x}_{3}}-1\le 0$$

$${g}_{2}\left(\overrightarrow{x}\right)=\frac{397.5}{{x}_{1}{x}_{2}^{2}{x}_{3}^{2}}-1\le 0$$

$${g}_{3}\left(\overrightarrow{x}\right)=\frac{1.93{x}_{4}^{3}}{{x}_{2}{x}_{3}{x}_{6}^{4}}-1\le 0$$

$${g}_{4}\left(\overrightarrow{x}\right)=\frac{1.93{x}_{5}^{3}}{{x}_{2}{x}_{3}{x}_{7}^{4}}-1\le 0$$

$${g}_{5}\left(\overrightarrow{x}\right)=\frac{\sqrt{{\left(\frac{745{x}_{4}}{{x}_{2}{x}_{3}}\right)}^{2}+16.9\times {10}^{6}}}{110.0{x}_{6}^{3}}-1\le 0$$

$${g}_{6}\left(\overrightarrow{x}\right)=\frac{\sqrt{{\left(\frac{745{x}_{4}}{{x}_{2}{x}_{3}}\right)}^{2}+157.5\times {10}^{6}}}{85.0{x}_{6}^{3}}-1\le 0$$

$${g}_{7}\left(\overrightarrow{x}\right)=\frac{{x}_{2}{x}_{3}}{40}-1\le 0$$

$${g}_{8}\left(\overrightarrow{x}\right)=\frac{{5x}_{2}}{{x}_{1}}-1\le 0$$

$${g}_{9}\left(\overrightarrow{x}\right)=\frac{{x}_{1}}{{12x}_{2}}-1\le 0$$

$${g}_{10}\left(\overrightarrow{x}\right)=\frac{{1.5x}_{6}+1.9}{{x}_{4}}-1\le 0$$

$${g}_{11}\left(\overrightarrow{x}\right)=\frac{{1.1x}_{7}+1.9}{{x}_{5}}-1\le 0$$

Variable interval:

$$2.6\le {x}_{1}\le 3.6 ,0.7\le {x}_{2}\le 0.8 ,17\le {x}_{3}\le 28 , 7.3\le {x}_{4}\le 8.3 ,7.8\le {x}_{5}\le 8.3,$$

$$2.9\le {x}_{6}\le 3.9, 5.0\le {x}_{7}\le 5.5$$

**Figure 7:**
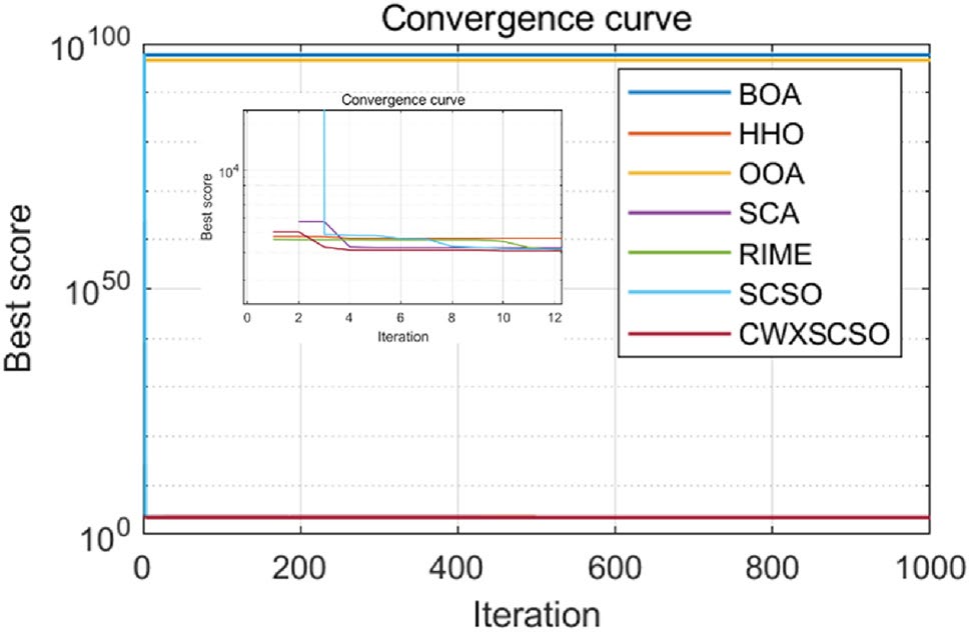
Reducer design optimization convergence curve.

Table 9 and Fig. 7 demonstrate that the modified method is adept at minimizing the weight of the reducer under 11 boundaries. It suggests that the enhancement is effective and may be more effectively utilized in mechanical systems.

**Table 9:** Optimization results of reducer design problems.

Algorithm	${x}_{1}$	${x}_{2}$	${x}_{3}$	${x}_{4}$	${x}_{5}$	${x}_{6}$	${x}_{7}$	Result
BOA	3.3994	0.7088	19.6705	7.8068	8.2178	3.4333	5.2689	1.9119E + 98
HHO	3.5827	0.7000	17.0000	8.2264	7.7636	3.4083	5.2867	3051.49
OOA	3.5411	0.7082	25.5903	7.7667	7.7708	3.5589	5.2860	4990.53
SCA	3.6000	0.7000	17.0000	7.6626	8.3000	3.6003	5.3054	3130.57
RIME	3.5003	0.7000	17.0000	7.3000	7.7227	3.3506	5.2868	2994.95
SCSO	3.5001	0.7000	17.0000	7.6154	8.1498	3.3510	5.2868	3007.17
CWXSCSO	3.5003	0.7000	17.0000	7.3000	7.7172	3.3503	5.2867	2994.66

### Step cone pulley problem

This engineering project aims to create a 4-step cone pulley with a minimal weight by looking at 5 design elements. Four variables represent the diameter of individual step of the pulley, denoted as ${d}_{i}(i=\mathrm{1,2},\mathrm{3,4})$, while the final variable represents the magnitude of the pulley's breadth, denoted as $w$. There are 8 nonlinear constraints and 3 linear constraints in the problem. The restriction is to maintain uniformity in the belt length ${C}_{i}$, tension ratio ${R}_{i}$, and belt transfer power ${P}_{i}$ throughout all steps. The mathematical description of the step cone pulley problem is as follows:

Function:

$$f\left(x\right)=\rho \omega \left[{d}_{1}^{2}\left\{1+{\left(\frac{{N}_{1}}{N}\right)}^{2}\right\}+{d}_{2}^{2}\left\{1+{\left(\frac{{N}_{2}}{N}\right)}^{2}\right\}+{d}_{3}^{2}\left\{1+{\left(\frac{{N}_{3}}{N}\right)}^{2}\right\}+{d}_{4}^{2}\left\{1+{\left(\frac{{N}_{4}}{N}\right)}^{2}\right\}\right]$$

Constraint condition:

$${h}_{1}\left(x\right)={C}_{1}-{C}_{2}=0, {h}_{2}\left(x\right)={C}_{1}-{C}_{3}=0 , {h}_{3}\left(x\right)={C}_{1}-{C}_{4}=0$$

$${g}_{\mathrm{1,2},\mathrm{3,4}}\left(x\right)={R}_{i}\ge 2, {g}_{\mathrm{5,6},\mathrm{7,8}}\left(x\right)={P}_{i}\ge \left(0.75*745.6998\right)$$

where:

$${C}_{i}=\frac{\pi {d}_{i}}{2}\left(1+\frac{{N}_{i}}{N}\right)+\frac{{\left(\frac{{N}_{i}}{N}-1\right)}^{2}}{4a}+2a i=\left(\mathrm{1,2},\mathrm{3,4}\right)$$

$${R}_{i}=exp\left[\mu \left\{\pi -2{{\text{sin}}}^{-1}\left\{\left(\frac{{N}_{i}}{N}-1\right)\frac{{d}_{i}}{2a}\right\}\right\}\right] i=\left(\mathrm{1,2},\mathrm{3,4}\right)$$

$${P}_{i}=stw\left[1-exp\left[-\mu \left\{\pi -2{{\text{sin}}}^{-1}\left\{\left(\frac{{N}_{i}}{N}-1\right)\frac{{d}_{i}}{2a}\right\}\right\}\right]\right]\frac{\pi {d}_{i}{N}_{i}}{60} i=\left(\mathrm{1,2},\mathrm{3,4}\right)$$

$$\rho =7200kg/{m}^{3} , a=3m ,\mu =0.35 ,s=1.75MPa ,t=8mm$$

Variable interval:

$$0\le {d}_{1}, {d}_{2}\le 60, 0\le {d}_{3}, \omega \le 90$$

**Figure 8:**
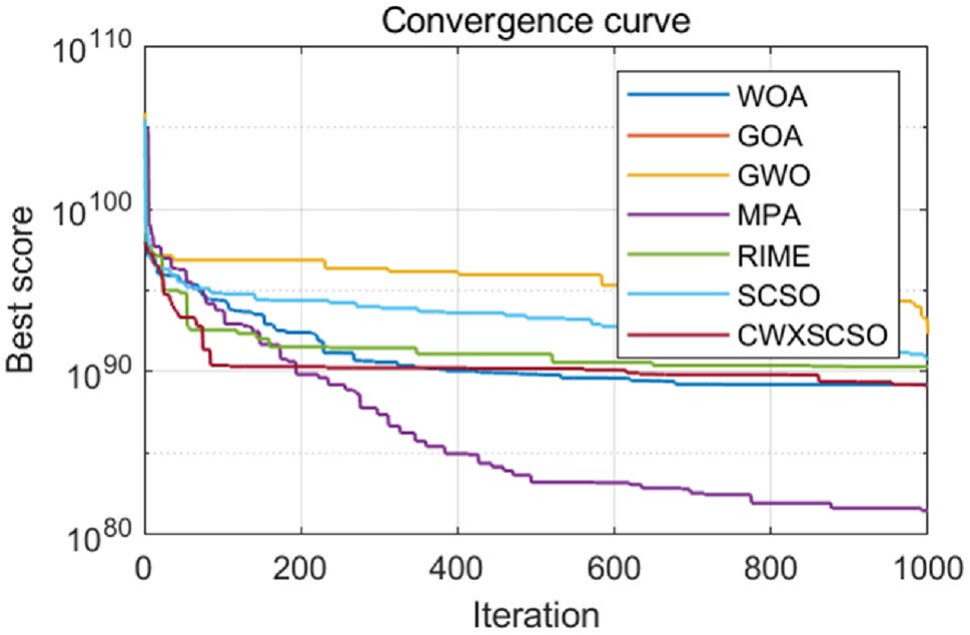
Optimization convergence diagram of step cone pulley problem.

Table 10 clearly demonstrates that the MPA method outperforms the CWXSCSO algorithm, but it still possesses certain advantages over other algorithms. Figure 8 illustrates that while the precision of convergence in CWXSCSO is less than that of MPA, its convergence speed beats that of MPA. Despite lacking MPA for the stepping cone pulley problem, CWXSCSO still has the benefit of rapid convergence speed.

**Table 10:** Optimization results of step cone pulley problem.

Algorithm	${d}_{1}$	${d}_{2}$	${d}_{3}$	${d}_{4}$	$w$	Result
WOA	41	56	75	90	85	1.6659E + 89
GOA	34	47	54	85	90	9.7914E + 97
GWO	41	56	74	89	89	2.2103E + 92
MPA	39	54	72	86	90	3.0726E + 81
RIME	39	54	72	87	90	2.0414E + 90
SCSO	40	56	74	89	86	5.3743E + 90
CWXSCSO	40	55	73	88	88	1.2490E + 89

### Planetary gear train design optimization problem

In power mechanical systems, the design of a planetary gear train presents a limited optimization problem. The issue encompasses three optimization variables, specifically the quantity of gear teeth $\left({N}_{1},{N}_{2},{N}_{3},{N}_{4},{N}_{5},{N}_{6}\right)$, gear modulus $\left({m}_{1},{m}_{2}\right)$, and the figure of merit $\left(p\right)$. The primary aim of the issue is to limit the maximum error associated with the transmission ratio employed in automotive production. The issue at hand encompasses a total of six integer variables, three discrete variables, and eleven distinct geometric and assembly restrictions. The mathematical description of the planetary gear train design optimization problem is as follows:

Variable:

$$x=\left({x}_{1},{x}_{2},{x}_{3},{x}_{4},{x}_{5},{x}_{6},{x}_{7},{x}_{8},{x}_{9}\right)=\left({N}_{1},{N}_{2},{N}_{3},{N}_{4},{N}_{5},{N}_{6},{m}_{1},{m}_{2},p\right)$$

Function:

$$f\left(x\right)=max\left|{i}_{k}-{i}_{ok}\right|, k=\left\{\mathrm{1,2},\dots ,R\right\}$$

where:

$${i}_{1}=\frac{{N}_{6}}{{N}_{4}}, {i}_{o1}=3.11, {i}_{2}=\frac{{N}_{6}\left({N}_{1}{N}_{3}+{N}_{2}{N}_{4}\right)}{{N}_{1}{N}_{3}\left({N}_{6}+{N}_{4}\right)}, {i}_{OR}=-3.11, {I}_{R}=-\frac{{N}_{2}{N}_{6}}{{N}_{1}{N}_{3}}, {i}_{O2}=1.84$$

Constraint condition:

$${g}_{1}\left(x\right)={m}_{2}\left({N}_{6}+2.5\right)-{D}_{max}\le 0$$

$${g}_{2}\left(x\right)={m}_{1}\left({N}_{1}+{N}_{2}\right)+{m}_{1}\left({N}_{2}+2\right)-{D}_{max}\le 0$$

$${g}_{3}\left(x\right)={m}_{2}\left({N}_{4}+{N}_{5}\right)+{m}_{2}\left({N}_{5}+2\right)-{D}_{max}\le 0$$

$${g}_{4}\left(x\right)=\left|{m}_{1}\left({N}_{1}+{N}_{2}\right)-{m}_{1}\left({N}_{6}+{N}_{3}\right)\right|-{m}_{1}-{m}_{2}\le 0$$

$${g}_{5}\left(x\right)=-\left({N}_{1}+{N}_{2}\right){\text{sin}}\left(\frac{\pi }{p}\right)+{N}_{2}+2+{\delta }_{22}\le 0$$

$${g}_{6}\left(x\right)=-\left({N}_{6}-{N}_{3}\right){\text{sin}}\left(\frac{\pi }{p}\right)+{N}_{3}+2+{\delta }_{33}\le 0$$

$${g}_{7}\left(x\right)=-\left({N}_{4}+{N}_{5}\right){\text{sin}}\left(\frac{\pi }{p}\right)+{N}_{5}+2+{\delta }_{55}\le 0$$

$${g}_{8}\left(x\right)={\left({N}_{3}+{N}_{5}+2+{\delta }_{35}\right)}^{2}-{\left({N}_{6}-{N}_{3}\right)}^{2}-{\left({N}_{4}+{N}_{5}\right)}^{2}+2\left({N}_{6}-{N}_{3}\right)\left({N}_{4}+{N}_{5}\right){\text{cos}}\left(\frac{2\pi }{p}-\beta \right)\le 0$$

$${g}_{9}\left(x\right)={N}_{4}-{N}_{6}+{2N}_{5}+2{\delta }_{56}+4\le 0$$

$${g}_{10}\left(x\right)={2N}_{3}-{N}_{6}+{N}_{4}+2{\delta }_{34}+4\le 0$$

$${h}_{1}\left(x\right)=\frac{{N}_{6}-{N}_{4}}{p}=integer$$

where:

$${\delta }_{22}={\delta }_{33}={\delta }_{55}={\delta }_{35}={\delta }_{56}=0.5$$

$$\beta =\frac{{cos}^{-1}\left({\left({N}_{4}+{N}_{5}\right)}^{2}+{\left({N}_{6}-{N}_{3}\right)}^{2}-{\left({N}_{3}+{N}_{5}\right)}^{2}\right)}{2\left({N}_{6}-{N}_{3}\right)\left({N}_{4}+{N}_{5}\right)}$$

$${D}_{max}=220$$

Variable interval:

$$P=\left(\mathrm{3,4},5\right), {m}_{1},{m}_{2}=\left(\mathrm{1.75,2.0,2.25,2.5,2.75,3.0}\right) , 17\le {N}_{1}\le 96,$$

$$14\le {N}_{2}\le 54, 14\le {N}_{3}\le 51, 17\le {N}_{4}\le 46, 14\le {N}_{5}\le 51, 48\le {N}_{6}\le 124$$

**Figure 9:**
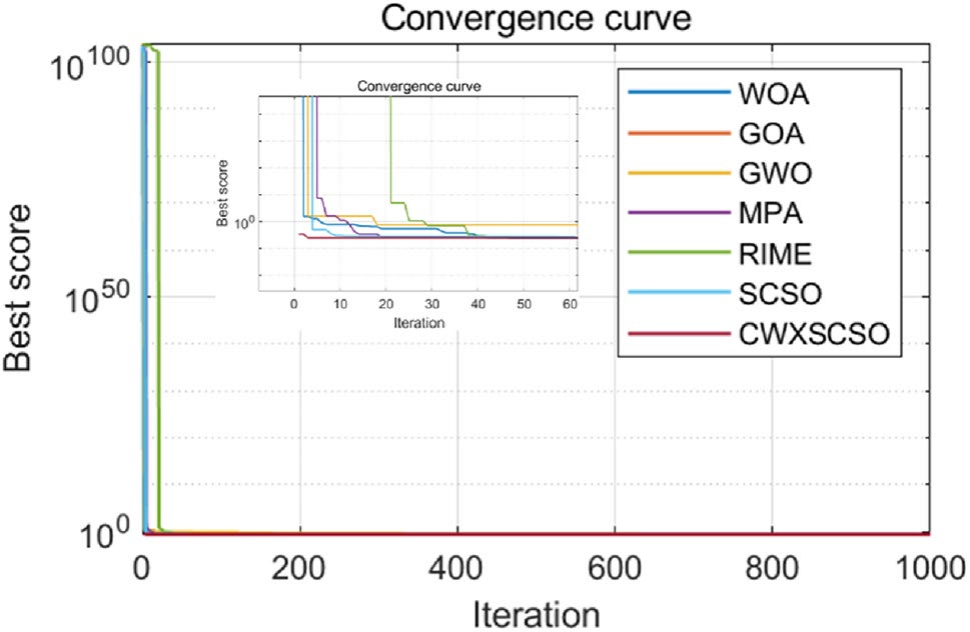
Convergence curve of planetary gear train design optimization problem.

Based on the data shown in Fig. 9 and Table 11, it is evident that CWXSCSO continues to outperform other methods in terms of convergence accuracy and convergence speed. This illustrates the potential for widespread implementation and utilization of the upgraded algorithm in power machinery.

**Table 11:** Results of planetary gear train design optimization problem.

Algorithm	${N}_{1}$	${N}_{2}$	${N}_{3}$	${N}_{4}$	${N}_{5}$	${N}_{6}$	${m}_{1}$	${m}_{2}$	$p$	Result
WOA	26	24	19	24	20	69	1	1	1	0.2422
GOA	17	16	14	17	14	50	2	1	1	0.2513
GWO	43	35	19	27	15	78	1	3	1	0.2379
MPA	30	24	22	32	22	92	3	1	1	0.2355
RIME	18	16	17	22	14	64	4	1	1	0.2398
SCSO	26	20	16	24	14	69	2	1	1	0
CWXSCSO	17	16	14	17	14	50	3	2	1	0

### Robot clamping optimization problem

The issue of robot hand claws is a complex challenge within the field of mechanical structure engineering. The goal of the robot clamping optimization is to minimize the disparity between the highest and lowest magnitudes of forces. The challenge of robot grippers encompasses a total of seven continuous design variables– the three connecting rods $(a,b,c)$, the vertical displacement of the linkages $(d)$, the vertical distance separating the initial node of the robotic arm from the end of the actuator $(e)$, the displacement in the horizontal direction between the actuator end and the linkages node $(f)$, and the angle of the second and third linkages in a geometric context $\left(\rho \right)$. There appear a total of seven distinct limitations. The mathematical description of the robot clamping optimization problem is as follows:

Variable:

$$x=\left({x}_{1},{x}_{2},{x}_{3},{x}_{4},{x}_{5},{x}_{6},{x}_{7}\right)=\left(a,b,c,d,e,f,p\right)$$

Function:

$$f\left(x\right)=-\underset{z}{{\text{min}}}{F}_{k}\left(x,z\right)+\underset{z}{{\text{max}}}{F}_{k}\left(x,z\right)$$

Constraint condition:

$${g}_{1}\left(x\right)=-{Y}_{min}+y\left(\left(x\right),{Z}_{max}\right)\le 0$$

$${g}_{2}\left(x\right)=-y\left(\left(x\right),{Z}_{max}\right)\le 0$$

$${g}_{3}\left(x\right)={Y}_{max}-y\left(\left(x\right),0\right)\le 0$$

$${g}_{4}\left(x\right)=y\left(\left(x\right),0\right)-{Y}_{G}\le 0$$

$${g}_{5}\left(x\right)={l}^{2}+{d}^{2}-{\left(a+b\right)}^{2}\le 0$$

$${g}_{6}\left(x\right)={b}^{2}-{\left(a-d\right)}^{2}-{\left(l-{Z}_{max}\right)}^{2}\le 0$$

$${g}_{7}\left(x\right)={Z}_{max}-f$$

where:

$$\alpha ={cos}^{-1}\left(\frac{{a}^{2}+{g}^{2}-{b}^{2}}{2ag}\right)+\Phi , g=\sqrt{{f}^{2}+{\left(z-f\right)}^{2}}$$

$$\beta ={cos}^{-1}\left(\frac{{b}^{2}+{g}^{2}-{a}^{2}}{2ag}\right)-\Phi ,\Phi ={tan}^{-1}\left(\frac{d}{f-z}\right)$$

$$y\left(x,z\right)=2\left(l+d+c{\text{sin}}\left(\beta +p\right)\right)$$

$${F}_{k}=\frac{Pb{\text{sin}}\left(\alpha +\beta \right)}{2c{\text{cos}}\left(\alpha \right)}, {Y}_{min}=50, {Y}_{max}=100, { Y}_{G}=150, P=100$$

Variable interval:

$$0\le d\le 50, 100\le c\le 200, 10\le e,a,b\le 150, 1\le p\le 3.14, 100\le f\le 300$$

**Figure 10:**
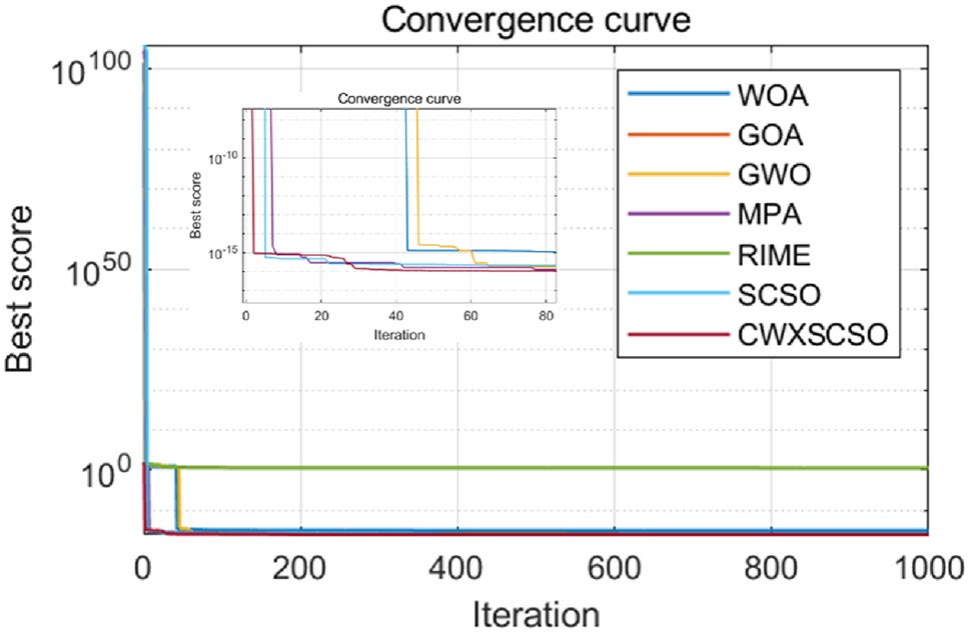
Convergence diagram of robot clamping optimization problem.

The data presented in Table 12 indicates that the CWXSCSO has the smallest disparity between its maximum force and minimum force. The curve convergence accuracy of CWXSCSO is the highest, as depicted in Fig. 10. Thus, the modified algorithm exhibits commendable competitive prowess within the field of mechanical engineering.

**Table 12:** Results of robot clamping optimization problem.

Algorithm	$a$	$b$	$c$	$d$	$e$	$f$	$\rho$	Result
WOA	100	38	100	0	10	100	1	1.4554E − 16
GOA	92	30	100	0	11	100	1	2.6670E − 16
GWO	100	38	199	0	125	100	2	7.3214E − 17
MPA	100	38	200	0	71	100	2	7.2741E − 17
RIME	100	38	200	0	101	100	2	7.3241E − 17
SCSO	100	38	200	0	14	100	1	7.0141E − 21
CWXSCSO	94	32	124	0	10	100	1	0

## Conclusion

The article introduces a sand cat swarm optimization algorithm which enhances the optimization power by including elite decentralization and crossbar technique. The algorithm that has been enhanced introduces a novel dynamic exponential factor. The position formula is updated using the elite decentralization technique, followed by the introduction of the crossbar strategy to accelerate the rate of convergence and improve the precision of search results. All strategies undergo testing using an assortment of 15 benchmark functions. CWXSCSO demonstrates superior search efficiency and stability, along with improved local search power and optimization accuracy, when compared to SCSO. Among the many strategies, elite decentralization and crossbar strategies have been found to be advantageous in enabling the algorithm beyond local optima. Simulation experiments were conducted on 10 test functions of CEC2019 and 10 test functions of CEC2021 using CWXSCSO and 6 other optimization algorithms. The results show that CWXSCSO outperforms the other 6 optimization algorithms in terms of optimization results. Additionally, CWXSCSO has the capacity to produce global optimal solutions for certain functions. The feasibility of the modified algorithm in practical engineering issues is further proven by the design of pressure vessel, welding beam, reducer, stepping cone pulley, planetary gear train design optimization, and robot clamping optimization. consequently, the primary purpose of this stage is to utilize it in order to address extensive, intricate multi-objective optimization problems and real engineering implementations.

### Supplementary Information

Supplementary Information.

## Data Availability

The datasets used and/or analysed during the current study available from the corresponding author on reasonable request.
