# Supplementary material for: Sand cat swarm optimization algorithm and its application integrating elite decentralization and crossbar strategy
Source: Sci Rep. 2024 Apr 18;14:8927. doi: 10.1038/s41598-024-59597-0 (PMC11026427; doi:10.1038/s41598-024-59597-0)
Supplement: Supplementary file 1 — Supplementary Information. [file 41598_2024_59597_MOESM1_ESM.docx]

Appendix A. Test function

A1. 23 benchmark functions

| function | Function name | dimension | Radius | Optimal value |
| --- | --- | --- | --- | --- |
| $f_{1}\left( x \right)=\sum_{i=1}^{n} x_{i}^{2}$ | Sphere Mode | 30 | [-100,100] | 0 |
| $f_{2}\left( x \right)=\sum_{i=1}^{n} \left\vert x_{i} \right\vert+\prod_{i=1}^{n} \left\vert x_{i} \right\vert$ | Schwefel’s problem 2.22 | 30 | [-10,10] | 0 |
| $f_{3}\left( x \right)=\sum_{i=1}^{n} \left( \sum_{j=1}^{i} x_{j} \right)^{2}$ | Schwefel’s problem 1.2 | 30 | [-100,100] | 0 |
| $f_{4}\left( x \right)={max}_{i}\left\{ \left\vert x_{i} \right\vert,1\leq i\leq n \right\}$ | Schwefel’s problem 2.21 | 30 | [-100,100] | 0 |
| $f_{5}\left( x \right)=\sum_{i=1}^{n-1} \left[ 100\left( x_{i+1}-x_{i}^{2} \right)^{2}+\left( x_{i}-1 \right)^{2} \right]$ | Generalized osenbrock’s Function | 30 | [-30,30] | 0 |
| $f_{6}\left( x \right)=\sum_{i=1}^{n} \left( \left[ x_{i}+0.5 \right] \right)^{2}$ | Step function | 30 | [-100,100] | 0 |
| $f_{7}\left( x \right)=\sum_{i=1}^{n} ix_{i}^{4}+random\left[ 0,\left. 1 \right) \right.$ | Quartic Function | 30 | [-1.28,1.28] | 0 |
| $F_{8}\left( x \right)=\sum_{i=1}^{n} -x_{i}\sin\left( \sqrt{\left\vert x_{i} \right\vert} \right)$ | Generalized Schwefel’s problem 2.26 | 30 | [-500,500] | -12569.5 |
| $F_{9}\left( x \right)=\sum_{i=1}^{n} \left[ x_{i}^{2}-10\cos\left( 2\pi x_{i} \right)+10 \right]$ | Generalized Rastrigin’s Function | 30 | [-5.12,5.12] | 0 |
| $F_{10}\left( x \right)=-20exp\left( -0.2\sqrt{\frac{1}{n}\sum_{i=1}^{n} x_{i}^{2}} \right)-exp\left( \frac{1}{n}\sum_{i=1}^{n} \cos\left( 2\pi x_{i} \right) \right)+20+e$ | Ackley’s Function | 30 | [-32,32] | 0 |
| $F_{11}\left( x \right)=\frac{1}{4000}\sum_{i=1}^{n} x_{i}^{2}-\prod_{i=1}^{n} \cos\left( \frac{x_{i}}{\sqrt{i}} \right)+1$ | Generalized Griewank Function | 30 | [-600,600] | 0 |
| $F_{12}\left( x \right)=\frac{\pi}{n}\left\{ 10\sin\left( \pi y_{1} \right)+\sum_{i=1}^{n-1} \left( y_{i}-1 \right)^{2}\left[ 1+10\sin^{2} \left( \pi y_{i+1} \right) \right]+\left( y_{n}-1 \right)^{2} \right\}+\sum_{i=1}^{n} u\left( x_{i},10,100,4 \right)$  $y_{i}=1+\frac{x_{i}+1}{4}$  $u\left( x_{i},a,k,m \right)=\left\{ \begin{aligned} k\left( x_{i}-a \right)^{m},x_{i}>a \\ 0,-a<x_{i}<a \\ k\left( {-x}_{i}-a \right)^{m},x_{i}<-a \end{aligned} \right.$ | Generalized Penalized Function | 30 | [-50,50] | 0 |
| $F_{13}\left( x \right)=0.1\left\{ \sin^{2} \left( 3\pi x_{i} \right)+\sum_{i=1}^{n} \left( x_{i}-1 \right)^{2}\left[ 1+\sin^{2} \left( 3\pi x_{1}+1 \right) \right]+\left( x_{n}-1 \right)^{2}\left[ 1+\sin^{2} \left( 2\pi x_{n} \right) \right] \right\}+\sum_{i=1}^{n} u\left( x_{i},5,100,4 \right)$ | Generalized Penalized Function | 30 | [-50,50] | 0 |
| $F_{14}\left( x \right)=\left( \frac{1}{500}+\sum_{j=1}^{25} \frac{1}{j+\sum_{j=1}^{2} \left( x_{i}-a_{ij} \right)^{6}} \right)^{-1}$ | Shekel’s Foxholes Function | 2 | [－65,65] | 1 |
| $F_{15}\left( x \right)=\sum_{i=1}^{11} \left[ a_{i}-\frac{x_{1}\left( b_{i}^{2}+b_{1}x_{2} \right)}{b_{i}^{2}+b_{1}x_{3}+x_{4}} \right]^{2}$ | Kowalik’s Function | 4 | [-5,5] | 0.0003075 |

A2. CEC2019 reference function

| No. | Function name | Search range | D | $F_{I}^{*}=F_{I}(x^{*})$ |
| --- | --- | --- | --- | --- |
| F1 | Storn's Chebyshev Polynomial Fitting Problem | [ -8192,8192] | 9 | 1 |
| F2 | Inverse Hilbert Matrix Problem | [ -16384,16384] | 16 | 1 |
| F3 | Lennard-Jones Minimum Energy Cluster | [ -4,4] | 18 | 1 |
| F4 | Rastrigin's Function | [ -100,100] | 10 | 1 |
| F5 | Griewangk's Function | [ -100,100] | 10 | 1 |
| F6 | Weierstrass Function | [ -100,100] | 10 | 1 |
| F7 | Modified Schwefel's Funetion | [- 100,100] | 10 | 1 |
| F8 | Expanded Schaffer's F6 Function | [- 100,100] | 10 | 1 |
| F9 | Happy Cat Function | [ -100,100] | 10 | 1 |
| F10 | Ackley Function | [ -100,100] | 10 | 1 |

A3. CEC2021 reference function

| No. | Function name | Search range | $F_{I}^{*}=F_{I}(x^{*})$ |
| --- | --- | --- | --- |
| C1 | Shifted and Rotated Bent Cigar Function | [ -100,100] | $100$ |
| C2 | Shifted and Rotated Schwefel’s Function | [ -100,100] | $1100$ |
| C3 | Shifted and Rotated Lunacek bi-Rastrigin Function | [ -100,100] | $700$ |
| C4 | Expanded Rosenbrock’s plus Griewangk’s Function | [ -100,100] | $1900$ |
| C5 | Hybrid Function 1 | [ -100,100] | $1700$ |
| C6 | Hybrid Function 2 | [ -100,100] | $1600$ |
| C7 | Hybrid Function 3 | [- 100,100] | $2100$ |
| C8 | Composition Function 1 | [- 100,100] | $2200$ |
| C9 | Composition Function 2 | [ -100,100] | $2400$ |
| C10 | Composition Function 3 | [ -100,100] | $2500$ |
